# Sacrificial Template‐Derived CoMo‐LDH Gas Diffusion Electrode for Anion Exchange Membrane Water Electrolysis

**DOI:** 10.1002/advs.202508370

**Published:** 2025-08-04

**Authors:** Sung Jun Lee, Youngtae Park, Seung Hun Lee, Seo Hyun Park, In Tae Kim, Youngji Kim, Baek San Soh, Geon Hwee Kim, Jooyoung Lee, Seunghwa Lee, Kihyun Shin, Yoo Sei Park

**Affiliations:** ^1^ Department of Urban, Energy, and Environmental Engineering Chungbuk National University Chungdae‐ro 1, Seowon‐Gu Cheongju Chungbuk 28644 Republic of Korea; ^2^ Hydrogen Research Department Korea Institute of Energy Research (KIER) 152 Gajeong‐ro, Yuseong‐gu Daejeon 34129 Republic of Korea; ^3^ Department of Chemical Engineering Changwon National University Changwon 51140 Republic of Korea; ^4^ Department of Materials Science and Engineering Pusan National University Busandaehak‐ro 63 beon‐gil 2, Geumjeong‐gu Busan 46241 Republic of Korea; ^5^ Department of Mechanical Engineering Chungbuk National University Chungdae‐ro 1, Seowon‐Gu Cheongju Chungbuk 28644 Republic of Korea; ^6^ Department of Materials Science and Engineering Hanbat National University Daejeon 34158 Republic of Korea; ^7^ Department of Energy & Environment Materials Research Division Korea Institute of Materials Science (KIMS) Changwon 51508 Republic of Korea; ^8^ Department of Nanoenergy Engineering Pusan National University Busandaehak‐ro 63 beon‐gil 2, Geumjeong‐gu Busan 46241 Republic of Korea; ^9^ Department of Nano Fusion Technology Pusan National University Busandaehak‐ro 63 beon‐gil 2, Geumjeong‐gu Busan 46241 Republic of Korea

**Keywords:** anion exchange membrane water electrolysis, electrocatalyst, gas diffusion electrode, in situ raman, oxygen evolution reaction, water splitting

## Abstract

Anion exchange membrane water electrolysis (AEMWE) offers a cost‐effective and efficient platform for hydrogen production by enabling the use of non‐platinum group metal (non‐PGM) electrode materials. However, the sluggish kinetics of the oxygen evolution reaction (OER) remains a key challenge. In this study, a CoMo‐LDH OER electrode for AEMWE is developed via a sacrificial template strategy. The high valence state of Mo promotes oxygen vacancy formation, enhancing OER performance. Electrochemical reconstruction also induces a phase transition into active (oxy)hydroxide species during OER. Density functional theory (DFT) calculations show that the weak OH^−^ adsorption energy of CoMo‐LDH lowers the energy barrier for OH^−^ deprotonation, improving catalytic activity. The CoMo‐LDH electrode demonstrates superior performance in AEMWE compared to the PGM‐based IrO_2_ electrode. This study highlights the potential of sacrificial template‐based electrodes for high‐performance AEMWE.

## Graphical abstract

This work presents high‐performance CoMo‐LDH electrodes for anion exchange membrane water electrolysis (AEMWE), developed via a sacrificial template method. High‐valence Mo induces oxygen vacancies and drives the lattice oxygen mechanism (LOM). In alkaline media, Mo leaches, forming active (oxy)hydroxide. This enhances oxygen evolution reaction (OER) activity toward non‐platinum group metal (PGM)‐based hydrogen production.

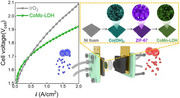

## 1 Introduction

Hydrogen has emerged as a highly efficient energy carrier and a promising alternative to fossil fuels, owing to its high energy density and clean combustion.^[^ ^1^ ^]^ To date, reforming has remained the predominant method for hydrogen production; however, this process inevitably generates carbon‐based emissions, limiting its environmental sustainability. As a cleaner alternative, electrochemical water splitting has garnered considerable attention for its ability to produce green hydrogen using renewable electricity, without emitting carbon pollutants.^[^ ^2^, ^3^ ^]^ This process consists of two electrochemical half‐reactions: the hydrogen evolution reaction (HER) at the cathode and the oxygen evolution reaction (OER) at the anode.^[^ ^4^, ^5^ ^]^ While the HER is directly responsible for hydrogen generation, the overall efficiency of water splitting is primarily limited by the sluggish kinetics of the OER, which involves complex multi‐electron transfer steps through hydroxyl intermediates. Therefore, the development of highly active OER electrocatalysts is crucial for improving the efficiency of water‐splitting systems and enabling the large‐scale production of green hydrogen.

Recently, the anion exchange membrane water electrolysis (AEMWE) has received attention as a cutting‐edge technology for water electrolysis. It allows a zero‐gap configuration, which minimizes the distance between the electrodes, thereby reducing internal cell resistance and improving efficiency.^[^ ^6^, ^7^ ^]^ Moreover, it operates in an alkaline environment, allowing the use of cost‐effective non‐platinum group metals (non‐PGMs) as electrode materials, which effectively reduces the cost of hydrogen production. Numerous OER electrodes have been developed for use in AEMWEs; however, their efficiency still lags behind that of proton exchange membrane water electrolyzers (PEMWEs).^[^ ^8^, ^9^ ^]^ Therefore, developing high‐performance OER electrodes for AEMWEs is essential to achieve high efficiency.

Layered double hydroxide (LDH), a hydrotalcite‐like compound, has attracted considerable attention as an electrocatalyst due to its theoretically high activity, excellent stability, and large surface area. The abundant coordination of unsaturated metal sites in LDH is particularly considered to lower the energy barrier for the oxygen evolution reaction (OER).^[^ ^10^, ^11^, ^12^ ^]^ However, the aggregation of LDH particles makes it difficult to form a uniform catalyst layer for the electrode, and its inherently low electrical conductivity poses a challenge for applying LDH in AEMWEs.^[^ ^13^ ^]^ One approach to address these issues is to fabricate an integrated electrode by growing LDH from its ionic state onto the electrode substrate. By growing LDH from its ionic state, the catalyst layer can grow uniformly on the electrode substrate. Among various methods for fabricating integrated electrodes, the sacrificial template approach allows for the straightforward formation of the LDH catalyst layer. Specifically, by chemically sacrificing the organic framework of a metal‐organic framework (MOF), LDH can be easily obtained.^[^ ^14^, ^15^ ^]^ MOFs are 3D structures formed by the coordination of metal ions with organic ligands. When the MOF structure is etched, its original framework is sacrificed, releasing metal ions while partially preserving the 3D morphology. By carefully controlling the etching conditions, the sacrificial process of the MOF can lead to the formation of LDH. Therefore, by forming a MOF on the electrode substrate and utilizing it as a sacrificial template, the development of an LDH‐based catalyst layer‐coated OER electrode for AEMWE becomes feasible.

Herein, we developed an OER electrode for AEMWE with a CoMo‐LDH catalyst layer coated on a commercial nickel foam (NF) using a sacrificial template strategy. Co(OH)_2_ was first deposited onto the NF via electrochemical co‐precipitation, followed by its conversion to zeolitic imidazolate framework‐67 (ZIF‐67) in a 2‐methylimidazole solution. Subsequently, the ZIF‐67 on NF was sacrificed in a solution containing Mo ions, leading to the formation of a CoMo‐LDH catalyst layer on the electrode. The incorporation of Mo with a high valence state promoted the generation of abundant oxygen vacancies, enhancing the OER performance. In addition, electrochemical reconstruction induced a phase transition into highly active (oxy)hydroxide species during the OER process. In situ Raman spectroscopy confirmed that the incorporation of Mo induces the formation of oxygen vacancies and suggested that CoMo‐LDH forms the active CoOOH phase at a lower potential compared to Co(OH)_2_, thereby contributing to enhanced OER activity. DFT calculations further revealed that the relatively weak OH^−^ adsorption energy of CoMo‐LDH lowers the free energy barrier for OH^−^ deprotonation, thereby contributing to its superior catalytic activity. Furthermore, the AEMWE equipped with the CoMo‐LDH electrode exhibited superior performance compared to the PGM‐based IrO_2_ electrode. This study demonstrates the potential of electrodes fabricated via a sacrificial template strategy as a viable approach for high‐performance AEMWE.

## 2 Results and Discussion

### 2.1 Characterization of CoMo‐LDH for Electrode Preparation

**Figure 1:**
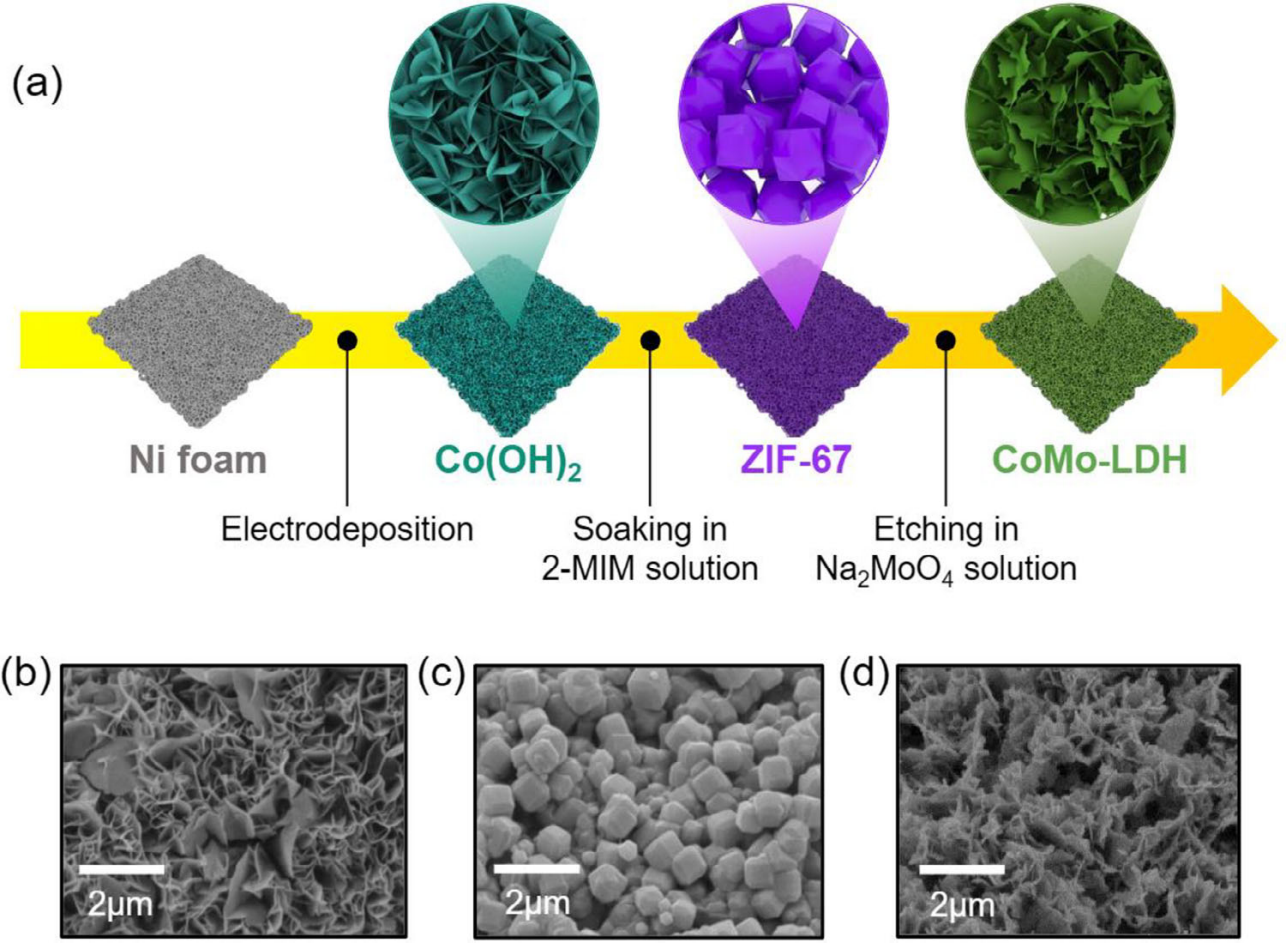
a) Schematic illustration for the preparation of CoMo‐LDH. SEM image of b) Co(OH)_2_, c) ZIF‐67 and d) CoMo‐LDH.

The CoMo‐LDH catalyst layer was prepared on a commercial nickel foam (NF) through a multistep process, as illustrated in **Figure** **1**a. Initially, the Co(OH)_2_ catalyst layer was coated on the NF via electrochemical co‐precipitation. Then, Co(OH)_2_ was transformed into ZIF‐67 by immersion in a 2‐MIM solution. A photograph of the prepared electrode is presented in Figure S1 (Supporting Information). Co(OH)_2_ exhibited a light blue color that shifted to deep purple upon immersion in the 2‐MIM solution. Subsequently, ZIF‐67 on NF was converted into CoMo‐LDH using the sacrificial template method, resulting in a light green color.

**Figure 2:**
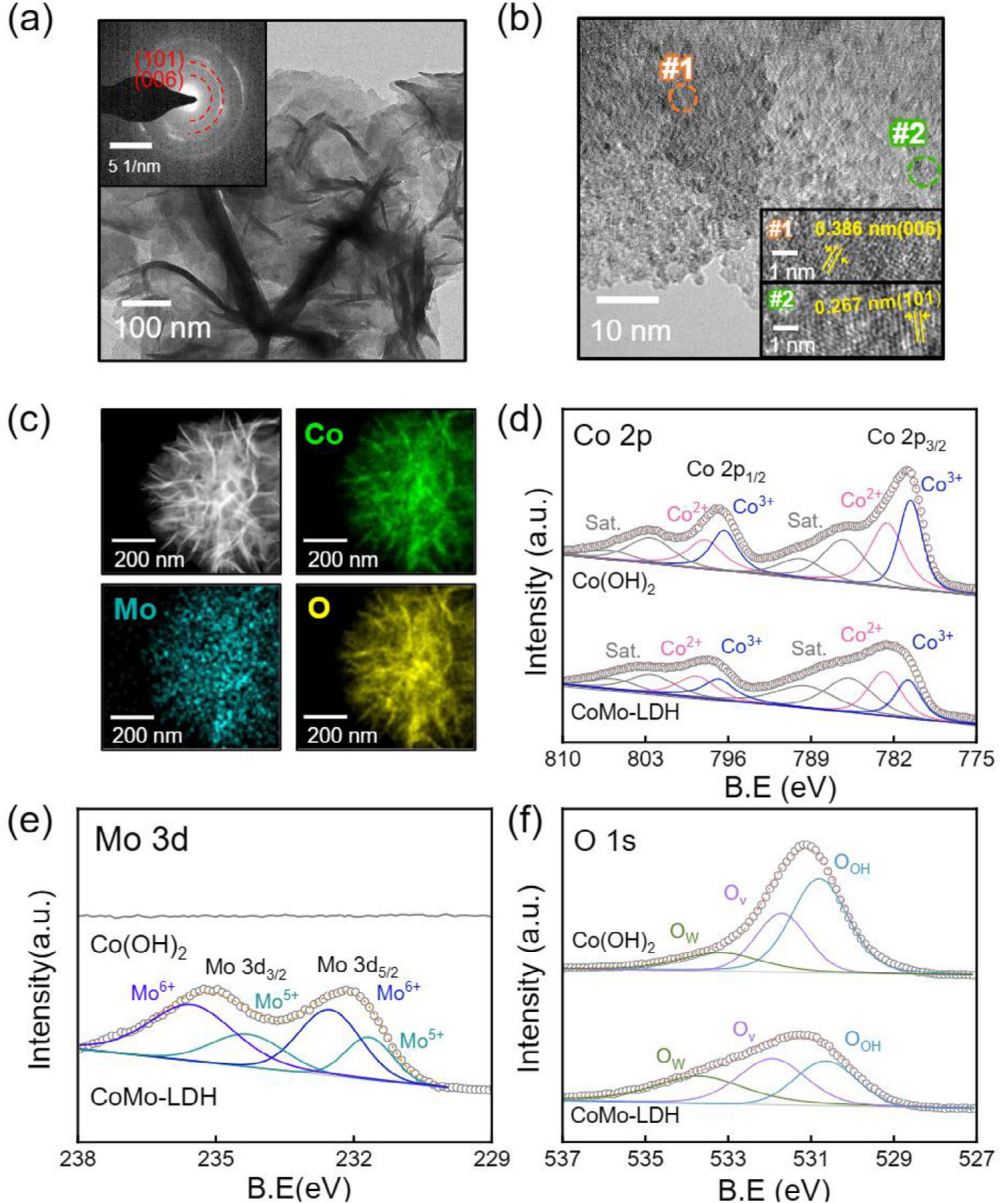
a) TEM image of the CoMo‐LDH with SAED pattern. b) HR‐TEM image of CoMo‐LDH. c) EDS elemental mapping images of the CoMo‐LDH: Co(green), Mo(blue), O(yellow). High‐resolution XPS spectrum of d) Co 2p, e) Mo 3d, and f) O 1s.

Surface morphologies of Co(OH)_2_, ZIF‐67, and CoMo‐LDH were analyzed using Field emission scanning electron microscopy (FE‐SEM), as shown in Figure 1b–d. Co(OH)_2_ exhibited nanosheet shapes, which transformed into the characteristic rhombohedral shape of ZIF‐67 after immersing in 2‐MIM solution.^[^ ^16^ ^]^ In comparison, CoMo‐LDH showed torn nanosheet shapes.^[^ ^17^ ^]^ The bulk crystal structures of the three prepared electrodes were confirmed through X‐ray diffraction (XRD) analysis, as shown in Figure S2a (Supporting Information). All samples showed peaks at 44.6°, 51.8°, and 76.4°, corresponding to metallic nickel (JCPDS: 00‐001‐1260), which is attributed to the nickel foam. However, no additional peaks apart from those for nickel were observed due to the thin catalyst layer. To obtain more detailed structural information, Grazing Incidence X‐ray Diffraction (GIXRD) analysis was performed in Figure S2b (Supporting Information). The GIXRD patterns of Co(OH)_2_ and CoMo‐LDH exhibited good agreement with the α‐Co(OH)_2_ (JCPDS:00‐046‐0605) and LDH (JCPDS: 00‐040‐215), respectively. To further investigate the shapes and crystal structure of Co(OH)_2_, ZIF‐67, and CoMo‐LDH, Transmission electron microscopy (TEM) images and selected area electron diffraction (SAED) patterns were analyzed. Consistent with the SEM images, Co(OH)_2_ showed nanosheets shapes, as shown in Figure S3 (Supporting Information), while ZIF‐67 exhibited its characteristic rhombohedral shape, as shown in Figure S4 (Supporting Information). CoMo‐LDH was observed to have a nanosheet shapes (**Figure** **2**a). The inset image reveals the SAED pattern of CoMo‐LDH, exhibiting a polycrystalline structure that aligns well with the (006) and (101) planes of LDH (JCPDS: 00‐040‐0215).^[^ ^18^ ^]^ Furthermore, HR‐TEM images of CoMo‐LDH, as shown in Figure 2b, showed d‐spacing values of 0.386 and 0.267 nm, corresponding to the (006) and (101) planes of LDH, respectively. The TEM‐Energy dispersive X‐ray spectroscopy (EDS) mapping images of Co(OH)_2_ and ZIF‐67, presented in Figures S5, S6 (Supporting Information), respectively, demonstrated a uniform distribution of atoms. Similarly, the atomic distribution in CoMo‐LDH was also uniform, as shown in Figure 2c. To conduct further analysis of the structure, Raman spectrum was obtained, as shown in Figure S7 (Supporting Information). The Raman spectrum of ZIF‐67 showed typical bands reported in previous studies.^[^ ^19^, ^20^ ^]^ The spectrum of Co(OH)_2_ showed 455 and 520 cm^−1^ vibrational modes, which were assigned to Co─OH and Co─O vibrations, respectively.^[^ ^21^, ^22^ ^]^ Notably, for CoMo‐LDH, the relatively broad bands observed ≈685 and 1050 cm^−1^ correspond to CO_3_^2−^ and NO_3_^−^_,_ indicating the presence of these interlayer anions and suggesting a double‐layer structure.^[^ ^23^, ^24^ ^]^ Furthermore, the bands observed in the region of 850–900 cm^−1^ correspond to Molybdenum, consisting of bands at 854 cm^−1^ (O─Mo─O) and 896 cm^−1^ (Mo─O), confirming the introduction of molybdenum.^[^ ^25^ ^]^ The Fourier transform infrared spectroscopy (FT‐IR) spectrum of CoMo‐LDH was also analyzed to verify its interlayer anions, as shown in Figure S8 (Supporting Information). The spectrum exhibited a significant absorption peak ≈3500 cm^−1^, indicative of O─H stretching, along with 1780 cm^−1^.^[^ ^26^ ^]^ Additionally, the absorption peaks observed in the range of 1250–1600 cm^−1^ correspond to the typical interlayer ion peaks of NO_3_^−^ and CO_3_^2−^, suggesting that NO_3_^−^ and CO_3_^2‐^ serves as interlayer ions in the LDH structure.^[^ ^27^ ^]^ Moreover, peaks within the extended wavelength range of 500–530 cm^−1^, along with a peak at 900 cm^−1^, indicate the presence of MoO_4_^2−^.^[^ ^28^ ^]^ Our research findings align well with previous studies, which reported that ZIF‐67 undergoes etching by water and Na_2_MoO_4_ precursor, leading to the release of Co^2+^ ions and the subsequent formation of CoMo‐LDH.^[^ ^17^ ^]^

To investigate the electronic structure, X‐ray photoelectron spectroscopy (XPS) spectrum of Co(OH)_2_ and CoMo‐LDH were obtained. The full XPS spectrum, shown in Figure S9 (Supporting Information), revealed a Mo peak at ≈235 eV in CoMo‐LDH.^[^ ^29^ ^]^ In the high‐resolution XPS spectrum of Co 2p, as shown in Figure 2d, two primary peaks corresponding to Co 2p_3/2_ and Co 2p_1/2_ were observed. These two main peaks were deconvoluted into Co^3+^ (≈780.6 eV) and Co^2+^ (≈782.6 eV) for Co(OH)_2_, respectively, whereas the corresponding peaks in CoMo‐LDH were deconvoluted into Co^3+^ (≈780.8 eV) and Co^2+^ (≈782.8 eV). The main peaks for CoMo‐LDH appeared at higher binding energies compared to those of Co(OH)_2_, signifying an increased ratio of Co^3+^ due to the incorporation of Mo. Additionally, notable changes were observed in the Co^3+^/Co^2+^ ratio, with values of 0.93 for Co(OH)_2_ and 0.82 for CoMo‐LDH. These results may be explained by the generation of oxygen vacancies induced by the incorporation of high‐valence Mo, as a means to maintain electrical neutrality.^[^ ^30^, ^31^, ^32^, ^33^ ^]^ In the high‐resolution XPS spectrum of Mo 3d for CoMo‐LDH, as shown in Figure 2e, two primary peaks corresponding to Mo 3d_5/2_ and Mo 3d_3/2_ were observed at 232.2 and 235.3 eV. Two deconvoluted peaks located at 232.5 and 235.6 eV are assigned to Mo^6+^, while the peaks at 231.6 and 234.3 eV are attributed to Mo^5+^.^[^ ^34^, ^35^, ^36^ ^]^ The high‐resolution XPS spectrum of O 1s, as shown in Figure 2f, revealed that both Co(OH)_2_ and CoMo‐LDH exhibit three types of oxygen species, corresponding to absorbed water (O_w_), oxygen vacancies (O_v_), and hydroxyl species (O_OH_). Notably, CoMo‐LDH exhibited a higher concentration of oxygen vacancies compared to Co(OH)_2_ in Figure S10 (Supporting Information). Oxygen vacancies enhance electronic conductivity, which contributes to improved electrode performance.^[^ ^31^, ^37^, ^38^, ^39^ ^]^

### 2.2 Electrochemical Analysis of Electrodes

**Figure 3:**
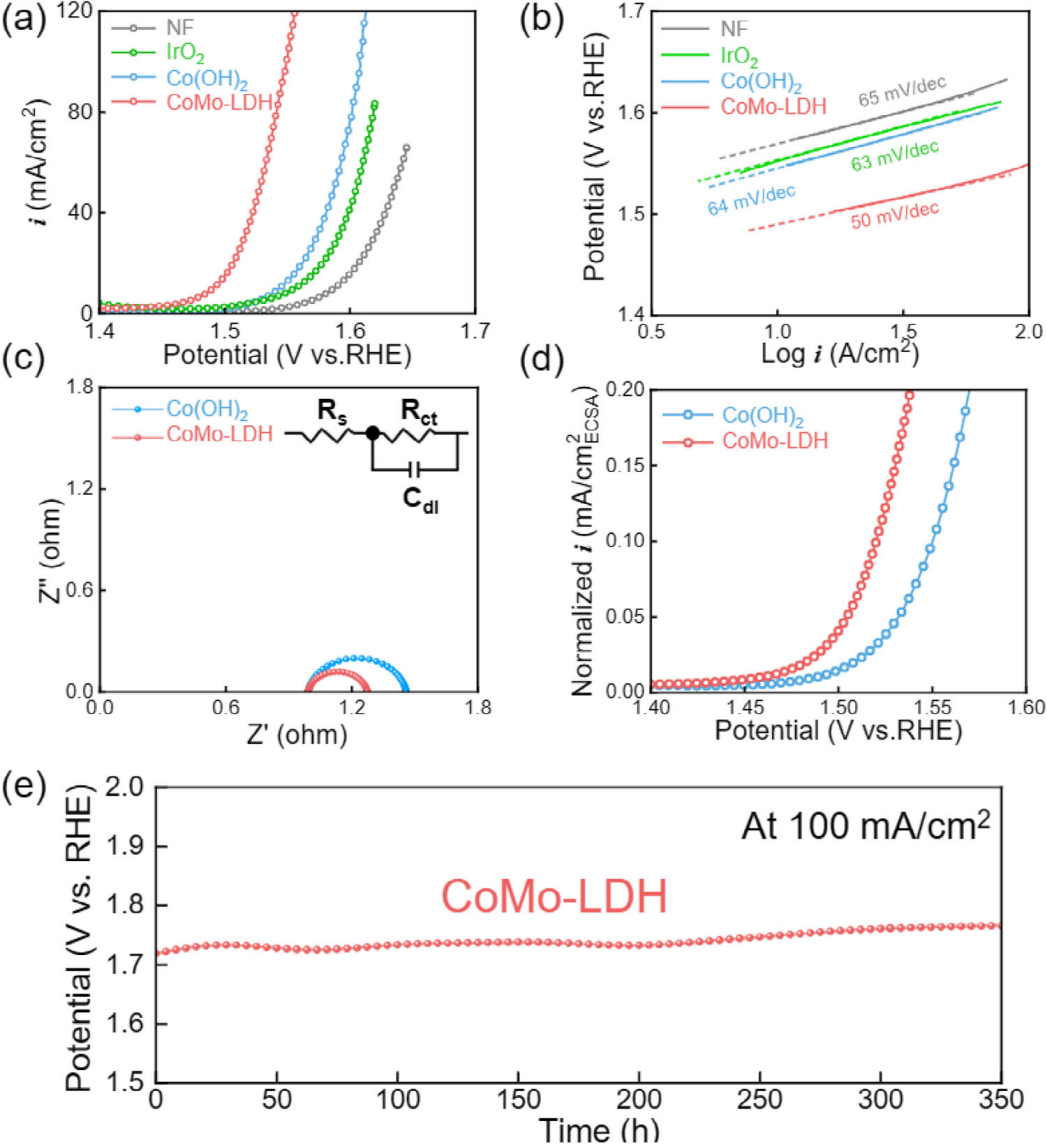
a) LSV polarization curves of NF, IrO_2_, Co(OH)_2,_ and CoMo‐LDH. b) corresponding Tafel plots and Tafel slopes of polarization curves. c) Nyquist plots of Co(OH)_2_ and CoMo‐LDH. d) Polarization curves of Co(OH)_2_ and CoMo‐LDH normalized by ECSA. e) Durability test of CoMo‐LDH at 100 mA cm^−2^ for 350 h.

To evaluate the electrode performance for OER, polarization curves and cyclic voltammetry curves were measured in a 1 m KOH solution, as shown in **Figures** **3**a and S11 (Supporting Information). The overpotential for OER was assessed at a current density of 10 mA cm^−2^. For comparison with PGMs‐based electrodes, an electrode coated with an IrO_2_ electrocatalyst on the NF was prepared. NF showed poor OER performance, with an overpotential of 350 mV, while IrO_2_ electrode showed enhanced OER performance, with an overpotential of 326 mV. Notably, CoMo‐LDH showed the best OER performance, recording an overpotential of 260 mV. Figure 3b presents Tafel plots, which indicate OER kinetics.^[^ ^40^ ^]^ The slopes of NF, Co(OH)_2,_ and IrO_2_ were similar, at ≈63 mV dec^−1^. In contrast, CoMo‐LDH demonstrated the lowest Tafel slopes of 50 mV dec^−1^, suggesting that the CoMo‐LDH had fast OER kinetics. Electrochemical Impedance Spectroscopy (EIS) analysis was performed, as shown in Figure 3c. The radius of the semi‐circles indicates charge transfer resistance (R_ct_).^[^ ^41^ ^]^ The R_ct_ values for Co(OH)_2_ and CoMo‐LDH were measured at 0.46 Ω and 0.28 Ω, respectively, demonstrating that CoMo‐LDH had faster charge transfer compared to Co(OH)_2_. To assess the surface roughness, the electrochemical active surface areas (ECSAs) were determined by measuring the double layer capacitance (C_dl_). Cyclic voltammetry (CV) measurements for Co(OH)_2_ and CoMo‐LDH were performed in the non‐faradaic region to calculate C_dl_, as shown in Figure S12 (Supporting Information).^[^ ^42^ ^]^ The C_dl_ values, shown in Figure S13 (Supporting Information), were 2.9 mF cm^−^^2^ for Co(OH)_2_ and 7.0 mF cm^−^^2^ for CoMo‐LDH, indicating that CoMo‐LDH had a higher surface roughness. The ECSAs, derived from C_dl_, were calculated to be 72.75 cm^2^ for Co(OH)_2_ and 175 cm^2^ for CoMo‐LDH, respectively. To evaluate the OER performance per ECSA, polarization curves of Co(OH)_2_ and CoMo‐LDH were normalized by ECSA, as shown in Figure 3d. CoMo‐LDH demonstrated superior OER performance per ECSA. Additionally, the Turnover Frequency (TOF), a critical indicator of catalytic activity, was determined for Co(OH)_2_ and CoMo‐LDH, as shown in Figure S14 (Supporting Information).^[^ ^43^ ^]^ TOF is calculated by dividing the amount of O_2_ gas generated by the number of metal active sites, assuming a 100% Faradaic efficiency.^[^ ^44^ ^]^ The TOF values were 0.007 O_2_ s^−1^ for CoMo‐LDH and 0.0005 O_2_ s^−1^ for Co(OH)_2_ at an overpotential of 300 mV, indicating that CoMo‐LDH produces O_2_ at a higher rate per metal site per second compared to Co(OH)_2_. To assess the durability of CoMo‐LDH, a chronopotentiometry test was performed at a current density of 100 mA cm^−^^2^, as shown in Figure 3e. The time‐dependent overpotential curve exhibited an increase of 7 mV over 400 h, indicating a degradation rate of 0.02 mV h^−1^. After the durability test, physical and chemical changes were analyzed by SEM image, Raman analysis, and XPS analysis, as shown in Figures S15–S17 (Supporting Information). Notably, the torn nanosheet observed in Figure 1d underwent surface reconstruction, resulting in a collapsed ZIF‐67. During prolonged oxidation reactions, modifications in the electronic structure of Co were observed, including an increase in the proportion of Co^3+^ following the OER.^[^ ^45^ ^]^ Notably, the peak associated with Mo disappeared, while a lattice oxygen (O_L_) species emerged, suggesting that Mo leached out during the OER, leading to the simultaneous formation of (oxy)hydroxide species, which are highly active for OER.^[^ ^46^ ^]^ To confirm the formation of (oxy)hydroxide species, the Raman spectrum of CoMo‐LDH before and after OER were compared, as shown in Figure S17 (Supporting Information). Consistent with the previous XPS results, the bands of Mo─O and O─Mo─O disappeared after the OER, indicating the leaching of Mo.^[^ ^47^ ^]^ Furthermore, the bands at 684 and 1055 cm^−1^, associated with interlayer anions, also disappeared, while new peaks emerged at 459, 508, and 655 cm^−1^, corresponding to E_g_ (CoO_2_), E_g_ (CoOOH), and (Co_3_O_4_), respectively.^[^ ^48^ ^]^ These peaks indicate the transformation of LDH to (oxy)hydroxide species during OER.^[^ ^49^ ^]^ Thus, these results demonstrated the formation of CoOOH, an active species for OER, due to the leaching of Mo during the OER.^[^ ^50^ ^]^

### 2.3 OER Mechanism

**Figure 4:**
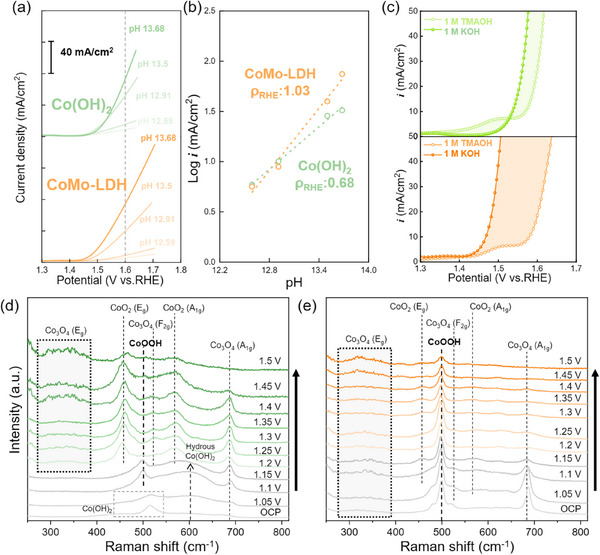
a) LSV curves for Co(OH)_2_ and CoMo‐LDH under varying pH conditions. b) Logarithmic plot of OER current density at 1.6 V versus RHE as a function of pH for proton reaction order (ρ_RHE_ = ∂log *i/*∂pH) calculation. c) LSV curves of Co(OH)_2_ and CoMo‐LDH in 1 m KOH and 1 m TMAOH. In situ Raman spectra of d) Co(OH)_2_ and e) CoMo‐LDH recorded under varying potentials from OCP to 1.5 V, with an increase of 0.05 V per step.

The previously observed significant difference in the Tafel slope of Co(OH)_2_ and CoMo‐LDH is noteworthy, as it may be attributed to differences in the competing OER mechanisms. To further explore the underlying cause of this observation, a pH‐dependence test was conducted in **Figure** **4**a. CoMo‐LDH exhibited a more pronounced enhancement in current density with increasing pH, compared to Co(OH)_2_. Based on these measurements, the proton reaction order (ρ_RHE_ = ∂log *i/*∂pH), which is an indicator of OER kinetic reaction for proton activity, was calculated to investigate the relationship between pH and OER activity, as shown in Figure 4b.^[^ ^51^, ^52^, ^53^, ^54^ ^]^ CoMo‐LDH exhibited a noticeably higher pH reaction order of 1.03, which is significantly greater than that of Co(OH)_2_ (0.68). A higher reaction order implies a stronger dependence of reaction kinetics on proton activity, which is likely due to the involvement of proton‐coupled electron transfer (PCET) step as the potential‐determining step (PDS).^[^ ^53^ ^]^ These observations are frequently observed in systems that proceed via the lattice oxygen mechanism (LOM).^[^ ^51^, ^52^, ^53^ ^]^ In the adsorbate evolution mechanism (AEM) pathway, lattice oxygen remains uninvolved, whereas in the LOM pathway, it actively participates in the reaction through oxidation and release, leading to the formation of oxygen vacancies.^[^ ^55^ ^]^ During this process, oxygen‐based intermediates such as O_2_^2−^ and O_2_^−^ play a pivotal role in driving the reaction forward. The tetramethylammonium cation (TMA^+^) can effectively scavenge oxygen‐based intermediates, thereby removing reactive oxygen species essential for the LOM mechanism and consequently suppressing the OER activity governed by LOM.^[^ ^51^, ^52^, ^53^, ^54^ ^]^ Figure 4c presented the OER performance of Co(OH)_2_ and CoMo‐LDH in 1 m KOH and 1 m TMAOH. Notably, the performance gap between the two electrolytes is significantly larger for CoMo‐LDH than Co(OH)_2_. This result suggests that CoMo‐LDH proceeds via the LOM pathway during OER, as TMA^+^ is likely to interact with O_2_^2‐^ intermediates, thereby interfering with the reaction involving lattice oxygen participation. The presence of oxygen vacancies in the LOM pathway weakens the surrounding M─O bonds and facilitates OH^−^ refilling.^[^ ^52^, ^53^ ^]^ This creates favorable sites for O─O coupling, thereby enhancing the OER activity. Therefore, this mechanism could be a contributing factor to the improved performance of CoMo‐LDH.

To monitor Mo dissolution from the CoMo‐LDH sample in real time, time‐resolved Raman spectroscopy was performed (Figure S18, Supporting Information). Upon exposure to 1 m KOH electrolyte, CoMo‐LDH exhibited a distinct spectral change compared to its ex situ Raman spectrum. A sharp peak corresponding to the A_1g_ mode of Co_3_O_4_ appeared dominantly near 685 cm^−1^, accompanied by additional minor peaks associated with Co_3_O_4_ observed ≈480 and 610 cm^−1^. In contrast, the Mo–O related peak near 900 cm^−1^, clearly observed under ex situ conditions, rapidly disappeared within 1 min of immersion, even without applying an external potential. This observation indicates that Mo dissolution proceeds spontaneously under highly alkaline conditions. Accompanied with the Mo dissolution, a gradual structural transformation of CoMo‐LDH was observed, wherein the material transitioned from Co_3_O_4_ to CoOOH over time. After ≈30 min of immersion, the spectral features of Co_3_O_4_ reached saturation, followed by the emergence of a broad yet distinct peak in the 300–350 cm^−1^ region, attributable to the E_g_ mode of Co_3_O_4_.^[^ ^56^ ^]^ A previous study has reported that this feature becomes pronounced at lower potentials under oxygen‐vacancy‐rich environments due to thermodynamically favorable OH^*^ adsorption.^[^ ^56^ ^]^ Based on this evidence, we assigned this spectral feature as a key indicator linked to oxygen vacancy formation. These findings motivated us to further investigate the dynamic phase transitions and associated oxygen vacancy evolution in Co(OH)_2_ and CoMo‐LDH under electrolytic conditions using in situ Raman spectroscopy.

Under alkaline conditions, Co‐based hydroxides can undergo a series of oxidation reactions with increasing applied potential as follow^[^ ^48^, ^57^ ^]^;

(1) $$3\mathrm{Co}{\left(\mathrm{OH}\right)}_{2}+2OH^{-}↔Co_{3}O_{4}+4H_{2}O\;+2e^{-}$$

(2) $$Co_{3}O_{4}+OH^{-}+H_{2}O↔3\mathrm{CoOOH}\;+e^{-}$$

(3) $$\mathrm{CoOOH}+OH^{-}↔\mathrm{Co}O_{2}+H_{2}O\;+e^{-}$$

The potential‐dependent phase transitions of Co(OH)_2_ and CoMo‐LDH are shown in Figure 4d,e. For Co(OH)_2_, characteristic peaks corresponding to Co–OH and Co–O vibrations are observed near 455 and 520 cm^−1^, respectively.^[^ ^58^ ^]^ As the potential increases, a broad band centered ≈600 cm^−1^ emerges, indicative of a transition to hydrous Co(OH)_2_.^[^ ^48^, ^58^ ^]^ Along with this change, additional peaks appear ≈500 and 685 cm^−1^, corresponding to the A_1g_ mode of Co_3_O_4_ and the formation of CoOOH.^[^ ^48^, ^57^, ^58^ ^]^ At potentials above 1.2 V, the formation of CoO_2_ becomes evident, accompanied by the emergence of a low‐frequency peak in the 300–350 cm^−1^ range, which is associated with the E_g_ mode of Co_3_O_4_ and serves as an indicator of oxygen vacancy formation. As the potential approaches the OER‐active region (≈1.4 V), this peak undergoes further evolution. Based on the spectra obtained at potentials above 1.45 V, we postulated that the active phases of Co(OH)_2_ under actual OER conditions consist of CoOOH along with more oxidized Co species, such as CoO_2_.

CoMo‐LDH (Figure 4e) exhibited a distinctly different potential‐dependent spectral behavior compared to Co(OH)_2_. The spectra revealed a sequential transformation involving Mo dissolution followed by the formation of Co_3_O_4_ and CoOOH. Note that both Co_3_O_4_ and CoOOH appeared at lower potentials relative to Co(OH)_2_, alongside the early emergence of the low‐frequency Co_3_O_4_ (E_g_) peak. These features collectively indicate that oxygen vacancies are preferentially generated in CoMo‐LDH under alkaline conditions. As the potential approached ≈1.25 V, the spectral contribution from Co_3_O_4_ diminished significantly, with CoOOH becoming the dominant phase, accompanied by a minor presence of CoO_2_. These results suggest that the active phases of CoMo‐LDH under OER conditions are similar to those of Co(OH)_2_, consisting primarily of CoOOH and more oxidized Co species such as CoO_2_, but with a higher concentration of oxygen vacancies derived from Mo dissolution during the structural transformation. The combination of abundant oxygen vacancies and the early formation of CoOOH at lower potentials likely contributed to the enhanced OER performance of CoMo‐LDH.^[^ ^56^, ^59^, ^60^ ^]^

### 2.4 DFT Calculation of CoMo‐LDH

First‐principle DFT calculations were performed to characterize Co(OH)_2_ and CoMo‐LDH, aiming to theoretically investigate their OER performance. DFT calculations were carried out using CoOOH as the structural model, which was identified as the active OER phase based on our Raman analysis. The CoOOH structure has been widely reported as the active phase derived from Co(OH)_2_ during the OER process, consistent with our experimental observations.^[^ ^49^, ^61^, ^62^ ^]^ Based on this structure, four different unit cell configurations with a 3:1 Co to Mo ratio were considered to identify the Mo site, and the lowest energy unit cell was selected for further analysis. Additionally, DFT calculations were conducted on the (011̅0) surface, commonly used to assess the catalytic activity of layered materials, as this surface is predominantly exposed at the edges of catalyst sheets.^[^ ^49^, ^62^, ^63^ ^]^ Consequently, the structures for comparing Co(OH)_2_ and CoMo‐LDH were successfully established.

In the experiment, the CoMo‐LDH demonstrated significantly higher OER performance than Co(OH)_2_. To confirm the influence of Mo, a theoretical approach was adopted. According to the Sabatier principle, which explains reaction kinetics, catalytic activity is determined by the relative adsorption energies of the OER intermediates.^[^ ^64^, ^65^, ^66^ ^]^ To estimate the OER activity, the following four‐electron reaction pathways were considered (Equations (4), (5), (6), (7)).

(4) $$\Delta G_{1}\;:\;4OH^{-}\left(\mathrm{aq}\right)↔OH^{*}+3OH^{-}\left(\mathrm{aq}\right)+e^{-}$$

(5) $$\begin{array}{cc} \Delta G_{2}:\;OH^{*}+3OH^{-}\left(\mathrm{aq}\right)+e^{-}↔\; & O^{*}+H_{2}O\left(l\right)+2OH^{-}\left(\mathrm{aq}\right)\\ & +\;2e^{-} \end{array}$$

(6) $$\begin{array}{cc} \Delta G_{3}:\;O^{*}+H_{2}O\left(l\right)+2OH^{-}\left(\mathrm{aq}\right)+2e^{-}↔\; & \mathrm{OO}H^{*}+H_{2}O\left(l\right)\\ & +OH^{-}\left(\mathrm{aq}\right)+3e^{-} \end{array}$$

(7) $$\begin{array}{cc} \Delta G_{4}:\;\mathrm{OO}H^{*}+H_{2}O\left(l\right)+OH^{-}\left(\mathrm{aq}\right)+3e^{-}↔\; & O_{2}\left(g\right)+2H_{2}O\left(l\right)\\ & +4e^{-} \end{array}$$

The overpotential (*η*) for OER was determined using the reaction energy diagram derived from the following equations (Equations 8 and 9)^[^ ^67^ ^]^:

(8) $$\Delta G\left(U\right)=\Delta E\;+\Delta \mathrm{ZPE}\;-T\Delta S\;-\mathrm{neU}$$

(9) $$\eta =U_{L}-1.23$$

In these equations, ∆E represents the ground‐state reaction energy, ∆ZPE accounts for the zero‐point energy correction, ∆S denotes the entropy change, U is the applied potential, and U_L_ is the limiting potential.^[^ ^68^, ^69^ ^]^

**Figure 5:**
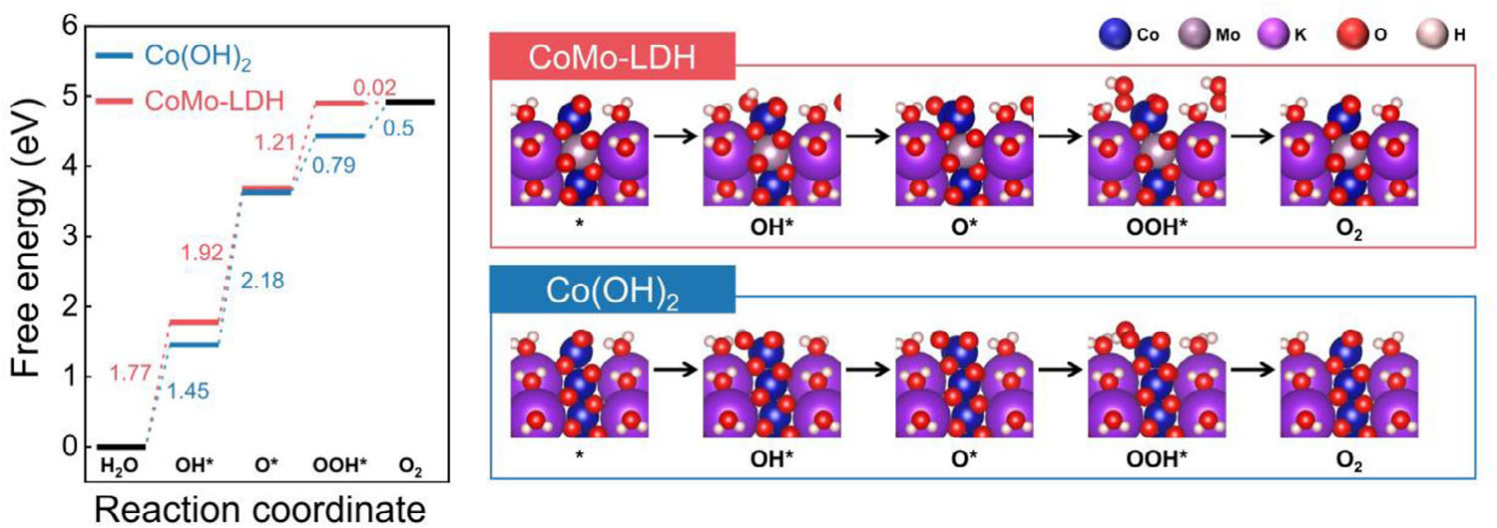
a) Gibbs free energy diagram for the four OER steps on Co(OH)_2_ and CoMo‐LDH. b) Most stable reaction intermediates on the Co(OH)_2_ and CoMo‐LDH surfaces: cobalt (blue), molybdenum (light purple), potassium (purple), oxygen (red), H (white).

Three key reaction intermediates (O^*^, OH^*^, and OOH^*^) were identified to construct a Gibbs free energy diagram for the OER mechanism. This study examined the OER mechanism on both Co(OH)_2_ and CoMo‐LDH. Optimized structures were obtained for these intermediates (O, OH, and OOH), and Gibbs free energy diagrams were constructed at U = 0, 1.23 V, and their respective limiting potentials, as shown in Figure S19 (Supporting Information). The PDS for both Co(OH)_2_ and CoMo‐LDH is the transformation of OH^*^ to O^*^, consistent with previous studies, resulting in overpotentials of 940 and 690 mV, respectively (**Figure** **5**).^[^ ^62^, ^69^ ^]^ Calculations indicate that the weakened OH adsorption energy in CoMo‐LDH, compared to Co(OH)_2_, reduces the free energy barrier for OH^*^ deprotonation, ultimately leading to higher catalytic activity in CoMo‐LDH, which aligns with experimental results.

### 2.5 AEMWE Testing

**Figure 6:**
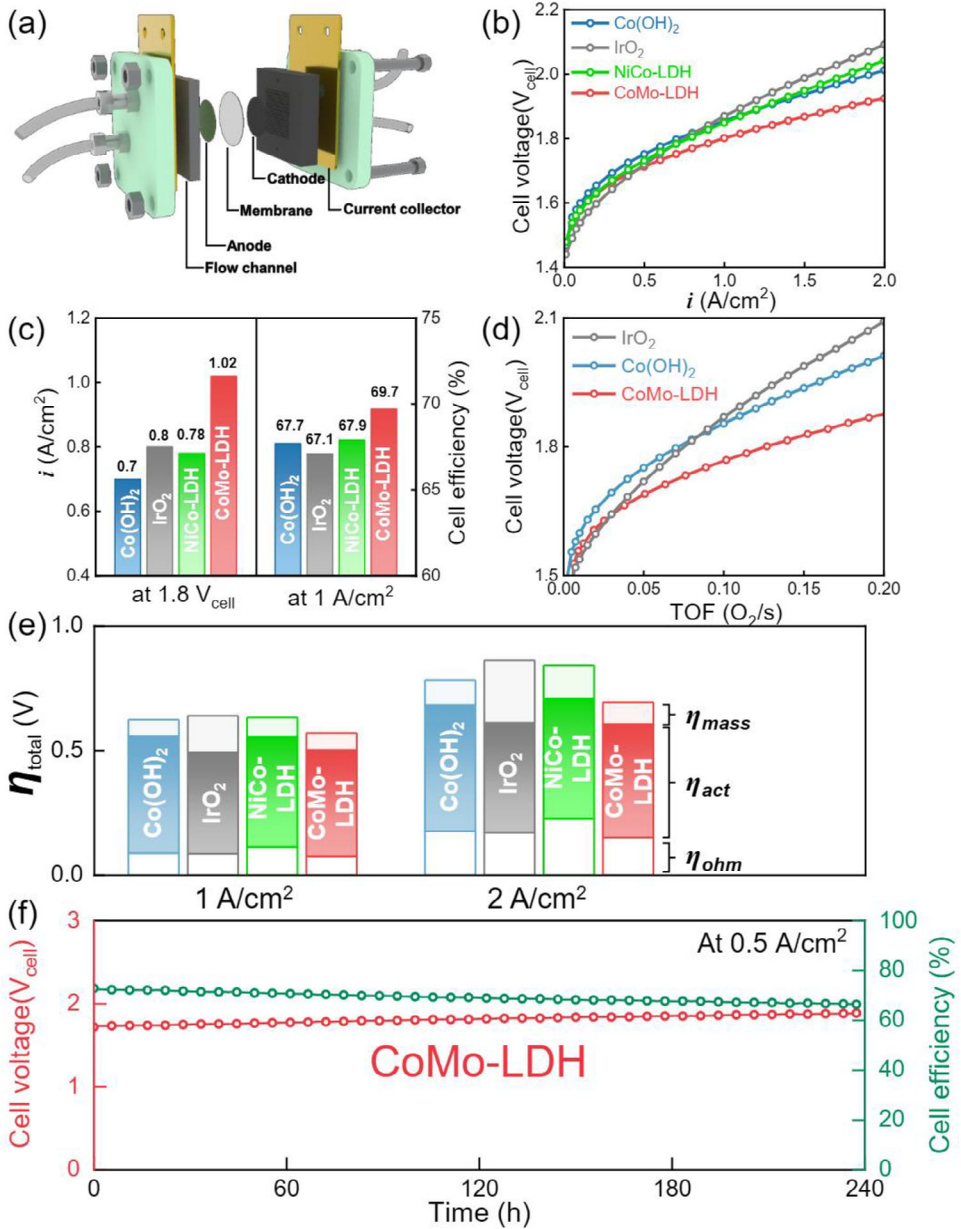
a) Schematic of AEMWE. b) The polarization curves of AEMWE. c) Current density at 1.8 V_cell_ and cell efficiency at 1 A cm^−2^. d) Turnover frequency (TOF). e) Overvoltage (*η*) subdivisions of the AEMWE by ohmic loss (*η*_ohm_), activation loss (*η*_act_), mass transport loss (*η*_mass_) at 1.0 and 2.0 A cm^−2^. f) Durability test and cell efficiency of AEMWE at 0.5 A cm^−2^.

To confirm practical applicability of prepared OER electrode, an AEMWE was assembled, consisting of an anode, cathode, anion exchange membrane (AEM), gold current collector, and flow channel, shown in **Figure** **6**a. For the HER electrode, Pt/C, known for its high performance among commercially available platinum group metal based HER electrocatalysts, was used to minimize performance loss.^[^ ^70^ ^]^ To compare with state‐of‐the‐art electrodes, NiCo‐LDH was fabricated using the electrodeposition method. For the OER electrode, IrO_2_, Co(OH)_2_, NiCo‐LDH, and CoMo‐LDH were employed. Polarization curves of AEMWE were obtained by applying a constant current density and measuring the cell voltage in Figure 6b. At 1.8 V_cell_, the AEMWE equipped with Co(OH)_2_ achieved a current density of ≈0.7 A/cm^2^, while IrO_2_ and NiCo‐LDH reached a current density of ≈0.8 and 0.78 A cm^−2^, respectively. The AEMWE equipped with CoMo‐LDH demonstrated the highest performance at a current density of ≈1.02 A cm^−2^, surpassing the PGM‐based OER electrode. Based on these polarization curves, cell efficiencies for the AEMWE were calculated as ≈67.7% (Co(OH)_2_), ≈67.1% (IrO_2_), ≈67.9% (NiCo‐LDH), and ≈69.7% (CoMo‐LDH), respectively, indicating higher efficiency than the PGM‐based electrode (Figure 6c). In addition, the TOF of the AEMWE was calculated (Figure 6d). At 1.8 V_cell_, TOF values were 0.07 O_2_ s^−1^ (Co(OH)_2_), 0.08 O_2_ s^−1^ (IrO_2_), and 0.125 O_2_ s^−1^ (CoMo‐LDH), indicating that CoMo‐LDH has significantly higher intrinsic activity in AEMWE.

To further examine performance difference across all voltage range, overvoltage was separated into ohmic loss (*η_ohmic_)*, activation loss (*η_act_*), and mass transport loss (*η_mass_*), as shown in Figure 6e and Figure S20 (Supporting Information). The ohmic losses of the AEMWE were similar at current densities of 1.0 and 2.0 A cm^−2^, indicating that other factors contribute to the variations in the overvoltage. Notably, CoMo‐LDH exhibited a lower activation loss (0.45 V) compared to Co(OH)_2_ (0.5 V) and NiCo‐LDH (0.48 V) at 2 A cm^−2^, suggesting that the AEMWE equipped with CoMo‐LDH had faster kinetics than that of Co(OH)_2_ and NiCo‐LDH.^[^ ^71^ ^]^ Although the polarization curve for the AEMWE equipped with CoMo‐LDH outperforms that of IrO_2_, the AEMWE with IrO_2_ exhibited the lowest activation loss, suggesting that AEMWE performance is influenced by factors beyond catalytic activity. Additionally, the AEMWE equipped with CoMo‐LDH showed similar mass transport loss to Co(OH)_2_, while demonstrating significantly lower mass transport loss than IrO_2_. The relatively higher mass transport loss observed in the AEMWE equipped with IrO_2_ at higher voltages may explain its rapid performance degradation in the high current density region. This behavior is attributed to the 3D nanosheet structure of Co(OH)_2_ and CoMo‐LDH, which facilitates the efficient removal of bubbles and reduces mass transport losses. To benchmark the performance of our AEMWE, Figures S21, S22, and Table S1 (Supporting Information) summarize the performance of AEMWEs employing non‐PGM based self‐supported anodes and cathode (Pt/C). The AEMWE equipped with CoMo‐LDH demonstrated superior performance compared to previously reported AEMWEs using self‐supported OER electrodes. To assess long‐term durability of the AEMWE equipped with CoMo‐LDH, a durability test was conducted at current density of 0.5 and 1.0 A cm^−2^, as shown in Figure 6f and Figure S23 (Supporting Information). Under harsh conditions, the AEMWE exhibited stable durability, with a deterioration rate was 700 µV h^−1^. Furthermore, the AEMWE maintained an efficiency exceeding 66.5% throughout the durability test, highlighting its robust performance. Faradaic efficiency was determined by measuring hydrogen generated during the test (Figure S24, Supporting Information), with the AEMWE equipped with CoMo‐LDH achieving a high faradaic efficiency of ≈98%, demonstrating excellent energy conversion. To investigate the structural and chemical changes in the electrode after AEMWE operation, a series of characterization analyses was conducted. Figure S25 (Supporting Information) exhibits SEM images of the CoMo‐LDH anode before and after durability test. It is worth noting that the post‐durability image shows that the morphology of CoMo‐LDH transformed into a nanosheet structure with a larger surface area. This structural change resulted from Mo leaching, which led to a surface reconstruction.^[^ ^35^, ^72^ ^]^ Additionally, as shown in Figure S26 (Supporting Information), the atomic ratio of Mo in CoMo‐LDH, as determined by SEM‐EDS, was significantly reduced after the durability test. Similar to the analysis in the half‐cell configuration, the Co 2p XPS spectrum of after durability test exhibited an overall negative shift compared to that before durability as shown in Figure S27a (Supporting Information). This shift indicates an increased proportion of Co^3+^ under the oxidative environment, suggesting the formation of CoOOH. Additionally, in Figure S27b (Supporting Information), the O 1s XPS spectrum revealed that the emergence of O_L_ peak at 529.9 eV, and the proportion of oxygen vacancies decreased from 50.1% to 19% after stability test. This strongly suggests a phase transition toward CoOOH, possibly accompanied by the consumption of oxygen vacancies along the reaction pathway. Finally, Figure S27c (Supporting Information) presents the Mo 3d XPS spectrum, demonstrating the surface dissolution of Mo following durability test. To evaluate economic feasibility, the electrical energy required to produce 1 kg of H_2_ under typical industrial‐scale conditions was calculated.^[^ ^73^ ^]^ As shown in Figure S28 (Supporting Information), the AEMWE equipped with CoMo‐LDH required 45.5 kWh to produce 1 kg of H_2_, which was lower than the energy demands of Co(OH)_2_ (46.6 kWh) and IrO_2_ (45.8 kWh). The CoMo‐LDH‐equipped AEMWE offers a cost advantage and superior performance, requiring less energy for commercial hydrogen production.

## 3 Conclusion

In this study, we fabricated CoMo‐LDH by converting hydroxide into the LDH structure using a sacrificial template method for the OER electrode. The CoMo‐LDH electrode exhibited exceptional activity (260 mV at 10 mA cm^−2^), outperforming both Co(OH)_2_ and the PGM‐based IrO_2_ electrode. This enhanced performance is attributed to the introduction of high‐valence molybdenum, which promotes the formation of oxygen vacancies and facilitates the formation of CoOOH during the OER. Importantly, in situ Raman spectroscopy revealed that the presence of oxygen vacancies in CoMo‐LDH facilitates the formation and stabilization of (oxy)hydroxide species at lower overpotentials, supporting its involvement in the LOM pathway. DFT analysis further confirmed that Mo in the CoMo‐LDH significantly enhances OER activity compared to Co(OH)_2_ by optimizing the adsorption energies of key intermediates. The AEMWE equipped with CoMo‐LDH demonstrated the highest performance (1.02 A cm^−2^ at 1.8 V_cell_) and cell efficiency (69.7% at 1.0 A cm^−2^), which can be ascribed to enhanced kinetics and improved mass transport. As a result, the system reduced the electrical energy required to produce 1 kg of hydrogen, highlighting its potential for both economic and practical advantages.

## 4 Experimental Section

### Chemical Reagents

Cobalt (II) nitrate hexahydrate (Co(NO_3_)_2_·6 H_2_O, ≥ 97.0%), potassium hydroxide (KOH, 95.0%), and hydrochloric acid (HCl, 35–37%) were sourced from Samchun, Korea. Sodium molybdate dihydrate (Na_2_MoO_4_·2 H_2_O, ≥ 95.0%) was supplied by Fisher Chemical, Korea. Daejung, Korea, provided 2‐methylimidazole (C_4_H_6_N_2_, ≥ 98%). A 5 wt.% Nafion solution and Iridium (IV) oxide powder (IrO_2_, 99.9%) were obtained from thermo scientific, while nickel foam was acquired from Welcos Co., Korea.

### Preparation of Co(OH)_2_ on NF

Cobalt hydroxide (Co(OH)_2_) was synthesized on NF using an electrochemical precipitation method. Initially, the surface oxide layers of NF were removed by immersing it in a 5 M hydrochloric acid (HCl) solution. The NF was then cleaned by deionized water (DI water) and ethanol. The synthesis of Co(OH)_2_ on the NF was carried out in a solution containing 50 mm Cobalt (II) nitrate hexahydrate (Co(NO_3_)_2_·6 H_2_O). The NF was used as a working electrode and titanium felt was used as a counter electrode. A saturated calomel electrode (SCE) was employed as the reference electrode. For electrodeposition, a potential of −1 V_SCE_ was applied for 5 min. Subsequently, the sample was rinsed with DI water and dried in an oven at 70 °C.

### Preparation of ZIF‐67 on NF

Zeolitic imidazolate framework‐67 (ZIF‐67) was synthesized through the phase transformation of Co(OH)_2_. The Co(OH)_2_, previously prepared on NF, was immersed in a solution containing 2‐methylimidazole for 24 h. This solution was prepared by dissolving 5 g of 2‐methylimidazole in 20 mL of DI water. After the 24‐h immersion, the sample was rinsed with DI water and subsequently dried in an oven at 70 °C.

### Preparation of CoMo‐LDH on NF

Cobalt‐Molybdenum‐Layered Double Hydroxide (CoMo‐LDH) was fabricated by etching ZIF‐67 in a solution containing sodium molybdate (Na_2_MoO_4_) at 85 °C for 30 min. This solution was prepared by dissolving 100 mg of Na_2_MoO_4_ in 25 mL of DI water. Following the immersion, the sample was thoroughly rinsed with DI water and dried in an oven at 70 °C.

### Preparation of NiCo‐LDH on NF

NiCo‐LDH was synthesized on NF using an electrochemical precipitation method.^[^ ^74^ ^]^ The oxide layer removal process for the nickel foam was identical to that used for Co(OH)_2_. The synthesis of NiCo‐LDH on the NF was conducted in a 50 mm mixed solution of Nickel (II) nitrate hexahydrate (Ni(NO_3_)_2_·6 H_2_O) and Cobalt (II) nitrate hexahydrate (Co(NO_3_)_2_·6 H_2_O) with a total molar ratio 1:2. The NF was used as a working electrode and titanium felt was used as a counter electrode. A saturated calomel electrode (SCE) was employed as the reference electrode. For electrodeposition, a potential of −1 V_SCE_ was applied for 5 min. Subsequently, the sample was rinsed with DI water and dried in an oven at 70 °C.

### Characterization

Surface morphologies were examined using field emission scanning electron microscopy (FESEM, SUPRA25) equipped with and energy‐dispersive X‐ray spectrometer. High‐resolution transmission electron microscopy (HRTEM) images were obtained using a Talos F200X (Thermo Fisher Scientific, USA). X‐ray diffraction (XRD) patterns were recorded on a X‐ray diffractometer (D8 VENTURE) at a scan speed of 2° min^−1^. X‐ray photoelectron spectroscopy (XPS) analyses were performed on a K‐Alpha (Multila‐2000) spectrometer. Fourier transform infrared (FT‐IR) spectrum were acquired using a Main Bench (Cary 670) equipped with a Cary 620 microscope in the range of 500–4500 cm^−1^ with a resolution of 0.06 cm^−1^. Raman spectrum was measured by XPER RAM S (Nano Base, South Korea).

### In Situ Raman spectroscopy

Raman spectroscopic measurements were conducted using a Raman microscope (XperRAM S, Nanobase) equipped with a 63x water immersion objective (Olympus) for both in situ and ex situ analyses. A transparent Teflon film (thickness: 0.001 in, McMaster‐Carr) was placed over the objective lens to prevent direct contact with the electrolyte solution. A 532 nm laser was used as the excitation source at a grating of 1800 l mm^−1^. The scattered light was collected by a charge‐coupled device (CCD) detector. The Raman shift was calibrated using the standard peaks of acetaminophen to ensure reproducibility and accuracy. Spectra were obtained with a resolution of ≈1 cm^−1^ by applying 30 consecutive scans with an exposure time of 0.7 s per scan at the laser spot. All Raman measurements were performed using a custom‐made electrochemical cell, with a platinum wire and a custom double‐junction Ag/AgCl electrode serving as the counter and reference electrodes, respectively. Prior to use, the cell was cleaned in an acid bath to remove residual metal ions and contaminants, followed by rinsing with acetone, ethanol, and deionized water.

### Electrochemical Analysis

Electrochemical measurements were carried out using a potentiostat (ZIVE MP1, WonaTech), with a three electrode system set up for the electrochemical analysis. The electrode (active size = 1 cm × 1 cm), was employed as the working electrode. A graphite rod was used as the counter electrode. The reference electrode was a Hg/HgO (1 m KOH). The measured potential was converted to a reversible hydrogen electrode (RHE) using the Nernst equation (E_RHE_ = E_Hg/HgO_ + 0.0591 × pH + 0.098). Polarization curves for OER were recorded at a scan rate of 1 mV s^−1^ with iR‐correction. Electrochemical surface areas (ECSAs) were determined using the equation: ECSAs = C_dl_/C_s_ (C_dl_: double layer capacitance, C_s_: capacitance of smooth plane metal surface). A durability test was performed at a current density of 100 mA cm^−2^ for 350 h. Electrochemical impedance spectroscopy (EIS) analysis was conducted at 1.677 V_RHE_ from 100 kHz to 1 Hz with an amplitude of 10 mV. Turnover frequency (TOF) was calculated using the formula: *i* / (4 × F × surface active sites), where *i* is the current density, F is the faraday constant. For comparison with PGMs‐based OER electrocatalyst, the IrO_2_ electrode was prepared (loading mass of IrO_2_ = 3 mg cm^−2^). The ink solution for the IrO_2_ was prepared by mixing IrO_2_ (20 mg), 5 wt.% Nafion solution (100 µL), and ethanol (900 µL). This mixture was then sonicated for 15 min. Subsequently, the resultant ink solution was drop‐coated onto the NF.

### AEMWEs Testing

The AEMWE consisted of anode (OER electrode), cathode (HER electrode), anion exchange membrane (AEM, SustainionX37‐50 Grade T, Dioxide Materials), flow channel, a gold‐plated for current collector, and an end plate. The cathode was fabricated by coating the ink on the carbon cloth with a microporous layer. The ink for cathode was obtained by mixing the isopropyl alcohol (IPA), DI water, Pt/C (40 wt.%, HISPEC 4000, Johnson Matthey) with 5 wt.% Nafion binder. The loading mass of Pt was 1.0 mg cm^−2^ ± 5%.

The CoMo‐LDH anode was fabricated by direct growth on the pressed nickel foam (NF). The ink solution for the IrO_2_ anode was synthesized by mixing the isopropyl alcohol, DI water, IrO_2,_ Nafion, and ionomer (Fumion FAA‐3‐solut‐10). The loading mass of IrO_2_ was 1.3 mg cm^−2^ ± 5%. The active area of the AEMWE was 2.54 cm^2^. The operating temperature was ≈55 °C and the electrolyte was supplied at a flow rate was 50 mL min^−1^. The polarization curves of the AEMWE were measured by DC power supply (MK‐W102, MKPOWER). Durability of the AEMWE was conducted by applying the constant current density of 0.5 A cm^−2^ for 233 h. The cell efficiency was calculated using the following equation.

(10) $$\mathrm{Cell}\;\mathrm{efficiency}\;\;\left(\%\right)=\frac{\Delta H_{H2,LHV}\;\times n_{H2,measured}\left(H_{2}power\right)}{I\times V\left(AEMelectrolyzerpower\right)}\times 100\%$$

where ΔH_H2,LHV_ is a lower heating value reaction enthalpy for water electrolysis (241.8 kJ mol^−1^), n_H2,measured_ is hydrogen production (mol s^−1^) measured by DC power supply, *I* is the applied current (A) and *V* is the applied voltage (V). The faradaic efficiency was computed using the following equation.

(11) $$\mathrm{FE}\;\left(\%\right)=\frac{nZF}{Q}\times 100$$

where n, Z, and F are the moles of electrons utilized to produce 1 mol of O_2_ or H_2_ generated, and the faraday constant, respectively. Q is the total quantity of utilized electricity. Typical industrial‐scale alkaline electrolyzers operate at a current density of 0.5 A cm^−2^, which is used to compute the electrical energy needed to produce 1 kg of hydrogen. Assuming the faradic efficiency for both the hydrogen evolution reaction and the oxygen evolution reaction processes is 100%, the amount of charge (Q) and electrical energy (W) required to generate 1 kg of H_2_ can be calculated using the following equation.

(12) $$Q\;=\frac{1000\;g\;\times N_{A}\times 2e^{-}}{MH_{2}\;\times \varepsilon }\;W\;=Q\;\times V$$

where N_A_ is Avogadro number, e^−^ is charge of an electron, M_H2_ is molecular mass of hydrogen ε is faradaic efficiency, and V is voltage of AEMWE at 0.5 A cm^−2^.

### Computational Details

The Vienna Ab initio Software Package (VASP) was utilized to conduct Density functional theory (DFT) calculations, employing the Perdew‐Burke‐Ernzerhof functional.^[^ ^75^ ^]^ The projector augmented wave (PAW) method with a plane wave energy cutoff of 520 eV was used to replace the interactions of core electrons. The criteria for electronic structure convergence and mean force were set to 3 × 10^−5^ eV and 0.05 eV Å^−1^, respectively. To account for correlation effects and mitigate self‐interaction errors, the Hubbard U‐parameter (DFT + U) was used with optimized effective interaction parameters (U_eff_ = U–J) of 3.32 and 4.38 eV for Co and Mo in Co(OH)_2_ and CoMo‐LDH, respectively.^[^ ^76^, ^77^, ^78^ ^]^ A vacuum thickness of 15 Å along the *z*‐axis was used to prevent surface interactions. During geometry relaxation, the top two layers of the slab were relaxed while the other two layers were fixed.

## Conflict of Interest

The authors declare no conflict of interest.

## Author Contributions

S.J.L., Y.P., and S.H.L. contributed equally to this work. All authors have given approval to the final version of the manuscript.

## Supporting information



Supporting Information

## Data Availability

The data that support the findings of this study are available from the corresponding author upon reasonable request.
