# Supplementary material for: Sacrificial Template‐Derived CoMo‐LDH Gas Diffusion Electrode for Anion Exchange Membrane Water Electrolysis
Source: Adv Sci (Weinh). 2025 Aug 4;12(40):e08370. doi: 10.1002/advs.202508370 (PMC12561300; doi:10.1002/advs.202508370)
Supplement: Supplementary file 1 — Supporting Information [file ADVS-12-e08370-s001.docx]

Supplementary Information

**Sacrificial Template-Derived CoMo-LDH Gas Diffusion Electrode for Anion Exchange Membrane Water Electrolysis**

*Sung Jun Lee^1,#^, Youngtae Park^2,#^, Seung Hun Lee^3,#^, Seo Hyun Park^1^, In Tae Kim^1^, Youngji Kim^4^, Baek San Soh^1^, Geon Hwee Kim^5^, Jooyoung Lee^6*^, Seunghwa Lee^4*^, Kihyun Shin^7*^, Yoo Sei Park^8,9*^*

^１^Department of Urban, Energy, and Environmental Engineering, Chungbuk National University, Chungdae-ro 1, Seowon-Gu, Cheongju, Chungbuk, 28644 Republic of Korea

^２^Hydrogen Research Department, Korea Institute of Energy Research (KIER), 152 Gajeong-ro, Yuseong-gu, Daejeon 34129, Republic of Korea

^3^ Department of Materials Science and Engineering, Pusan National University, 2 Busandaehak-ro 63beon-gil, Geumjeong-gu, Busan 46241, Republic of Korea

^4^ Department of Chemical Engineering, Changwon National University, 51140, Changwon, Republic of Korea

^5^ Department of Mechanical Engineering, Chungbuk National University, Chungdae-ro 1, Seowon-Gu, Cheongju, Chungbuk, 28644 Republic of Korea

^6^ Department of Energy & Environment Materials Research Division, Korea Institute of Materials Science (KIMS), Changwon, Republic of Korea

^7^ Department of Materials Science and Engineering, Hanbat National University, Daejeon, 34158 Republic of Korea

^8^ Department of Nanoenergy Engineering, Pusan National University, Busandaehak-ro 63 beon-gil 2, Geumjeong-gu, Busan 46241, Republic of Korea

^9^ Department of Nano Fusion Technology, Pusan National University, Busandaehak-ro 63 beon-gil 2, Geumjeong-gu, Busan 46241, Republic of Korea

# These authors contributed equally to this work.

**Email:**

[yoosei@pusan.ac.kr](mailto:yoosei@pusan.ac.kr) (Prof. Yoo Sei Park)

[kihyun@hanbat.ac.kr](mailto:kihyun@hanbat.ac.kr) (Prof. Kihyun Shin)

seunghwa@changwon.ac.kr (Prof. Seunghwa Lee)

ljy5424@kims.re.kr (Dr. Jooyoung Lee)


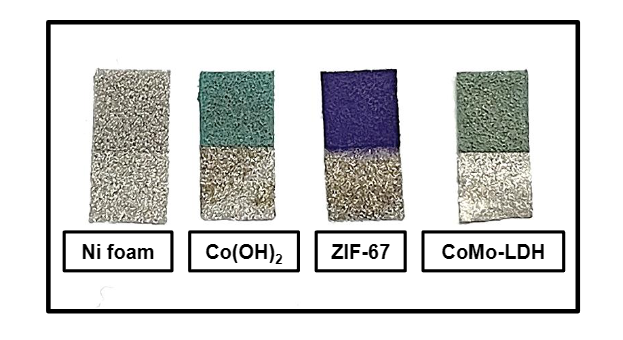


**Figure S1**. Photograph of Ni foam, Co(OH)_2_, ZIF-67 and CoMo-LDH.


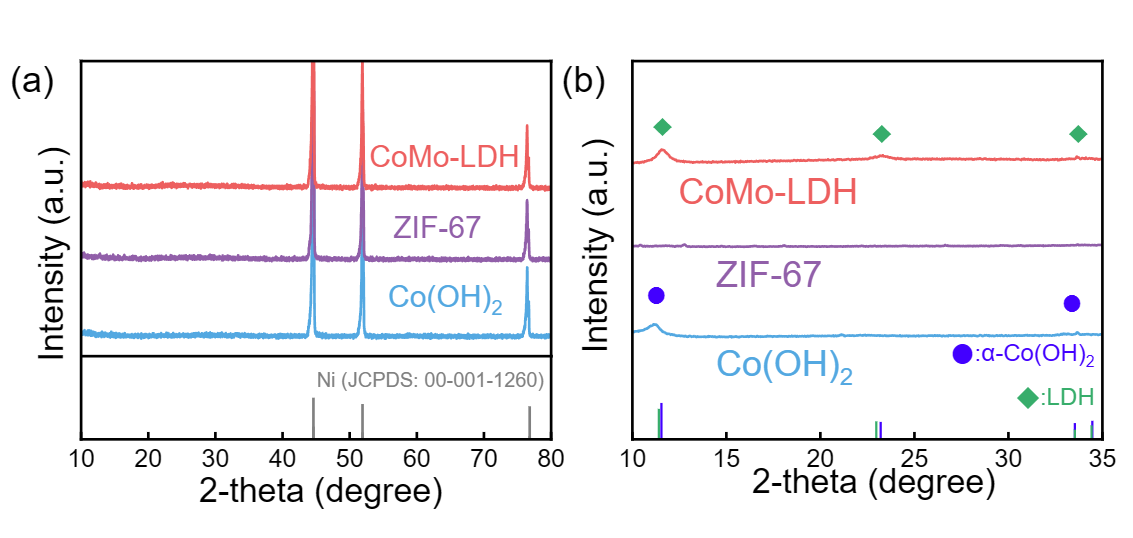


**Figure S2.** (a) XRD pattern of Co(OH)_2_, ZIF-67 and CoMo-LDH. (b) Grazing incidence X-ray Diffraction (GIXRD) patterns of Co(OH)_2_, ZIF-67 and CoMo-LDH.


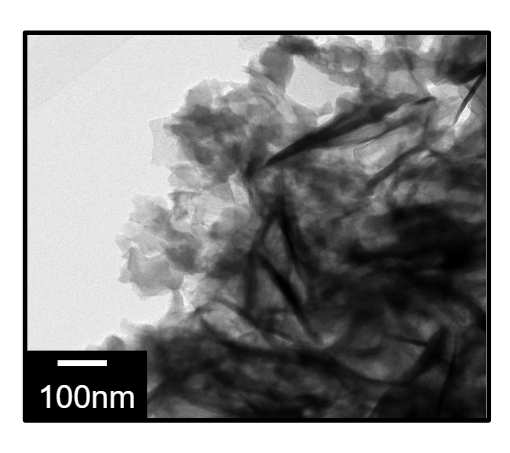


**Figure S3**. TEM image of Co(OH)_2_.


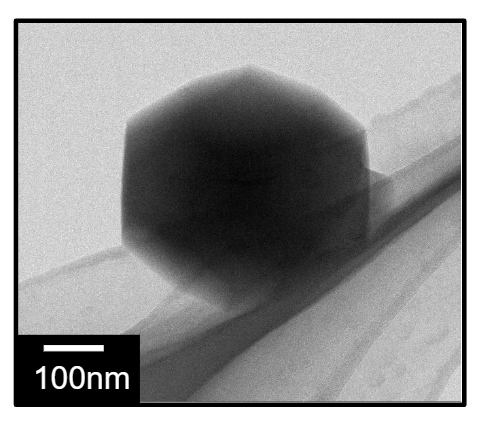


**Figure S4**. TEM image of ZIF-67.


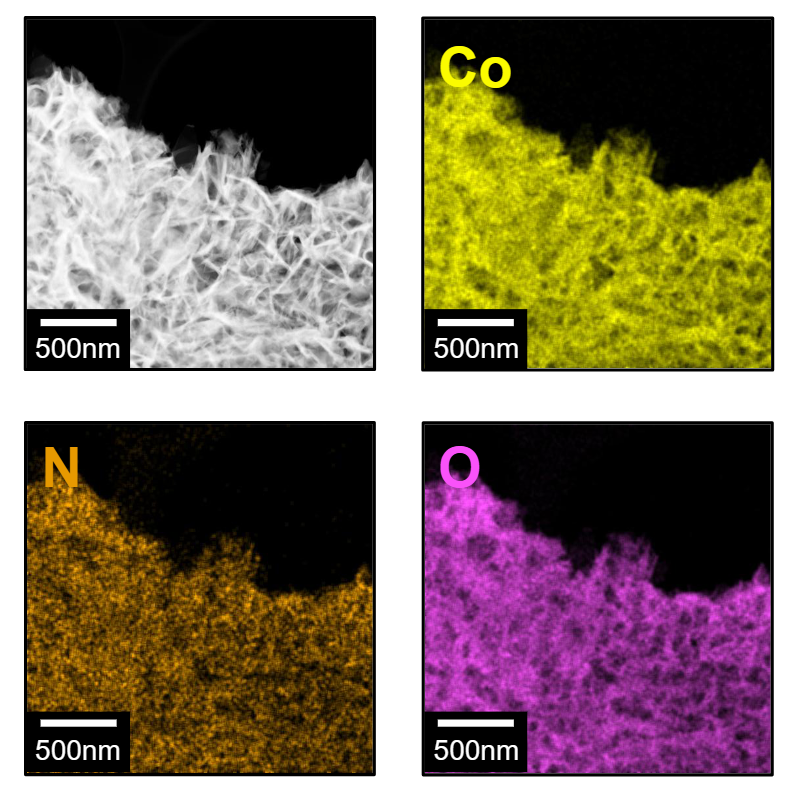


**Figure S5**. TEM-EDS mapping image of Co(OH)_2_.


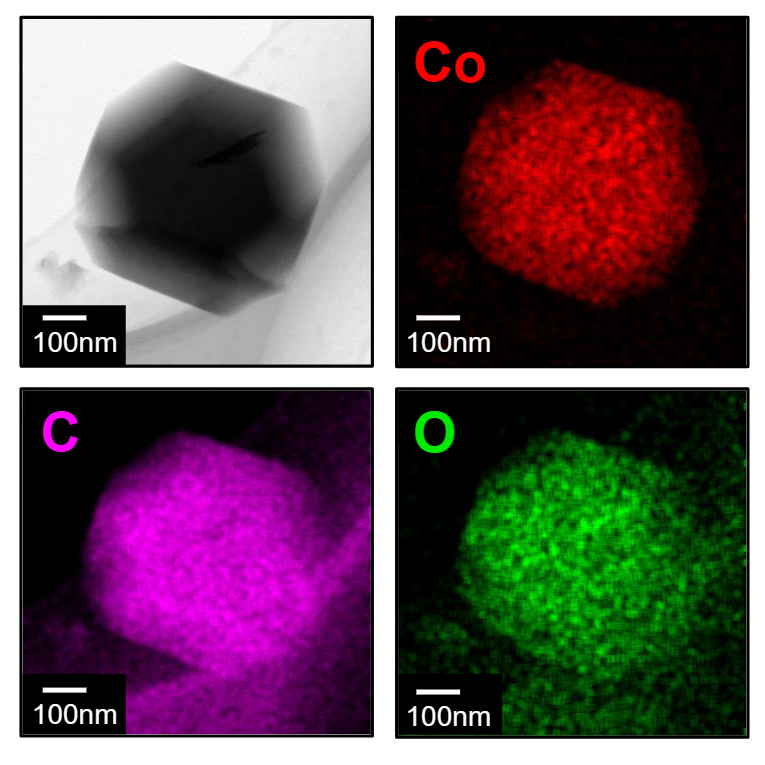


**Figure S6**. TEM-EDS mapping image of ZIF-67.


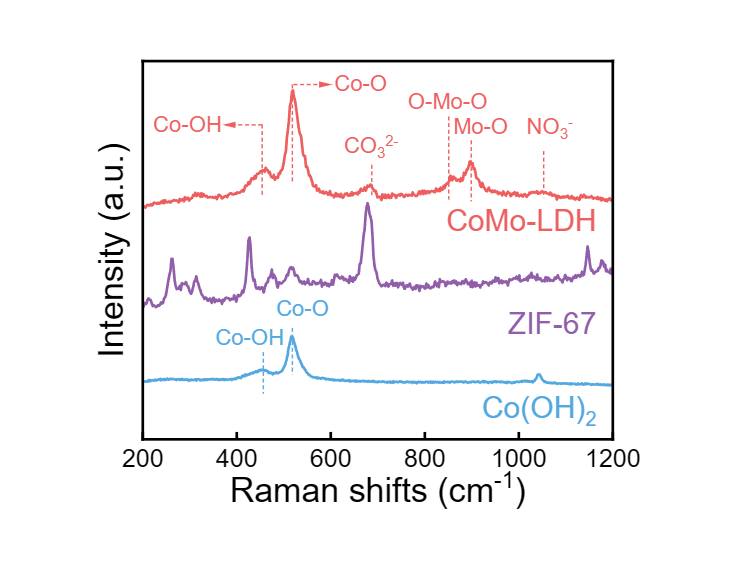


**Figure S7.** Raman spectrum of Co(OH)_2_, ZIF-67 and CoMo-LDH.


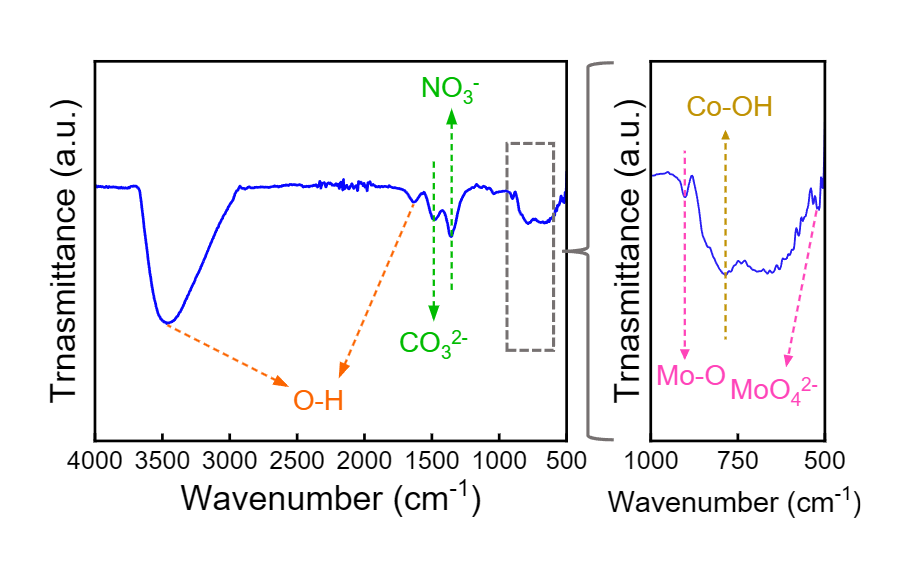


**Figure S8**. Fourier Transform Infrared Spectroscopy (FT-IR) spectra of CoMo-LDH.


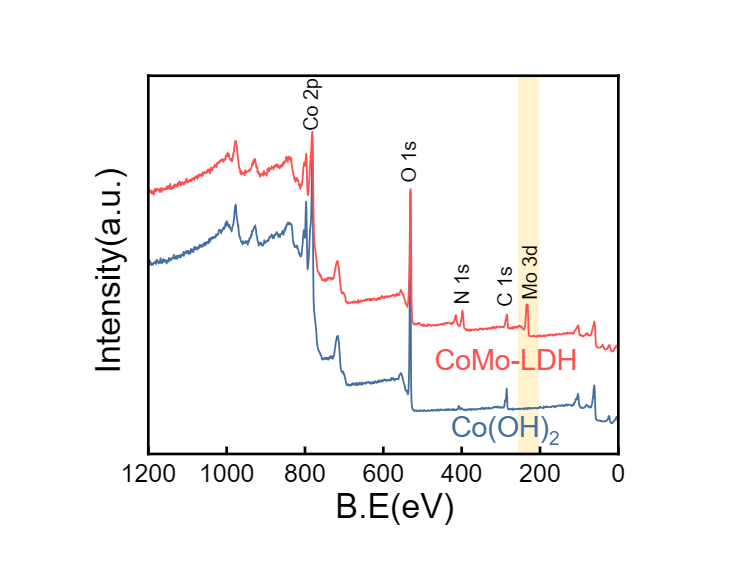


**Figure S9**. Full survey scan of Co(OH)_2_ and CoMo-LDH.


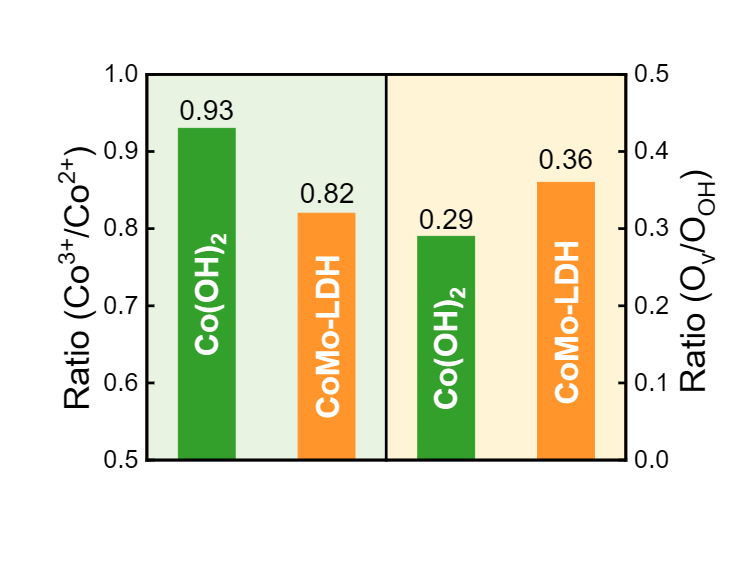


**Figure S10**. The ratio of Co^3+^/Co^2+^ and O_v_/O_OH_.


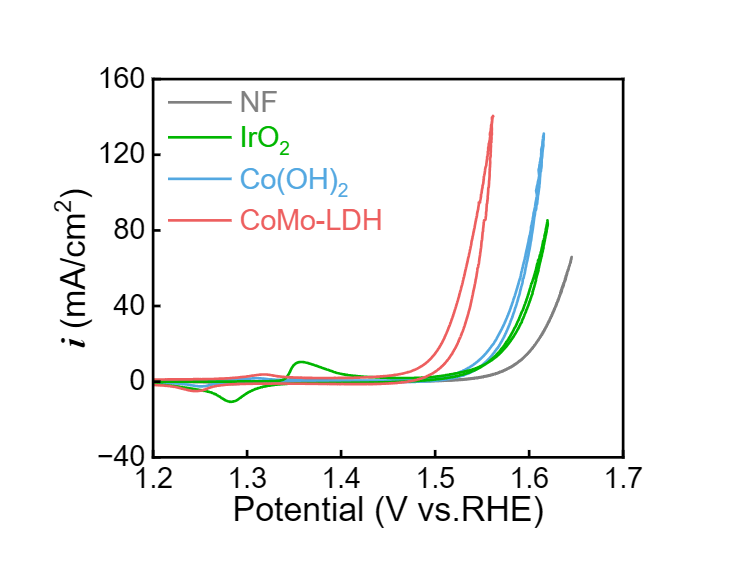


**Figure S11**. Cyclic voltammetry curves of NF, IrO_2_, Co(OH)_2_ and CoMo-LDH.


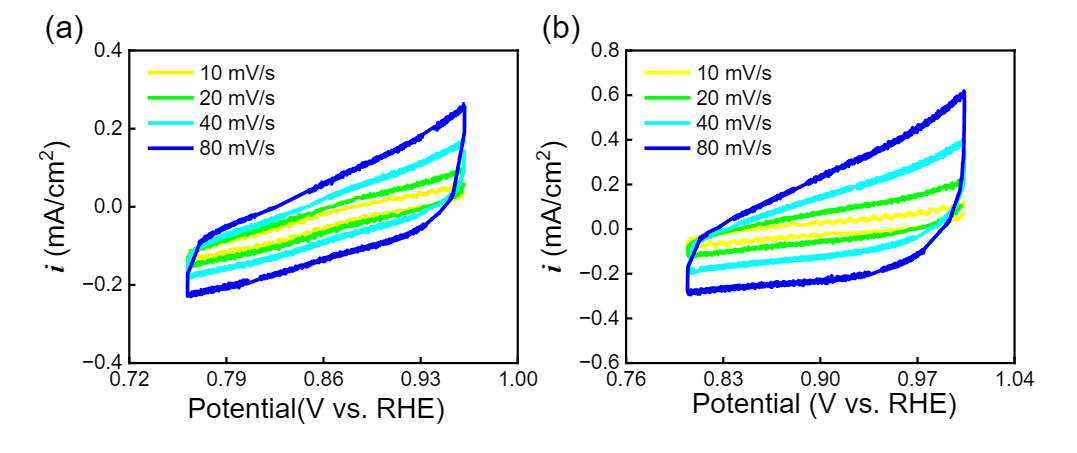


**Figure S12.** Cyclic voltammetry of (a) Co(OH)_2_ and (b) CoMo-LDH with a scan rate of 10, 20, 40, and 80 mV/s in the non-faradic regions.


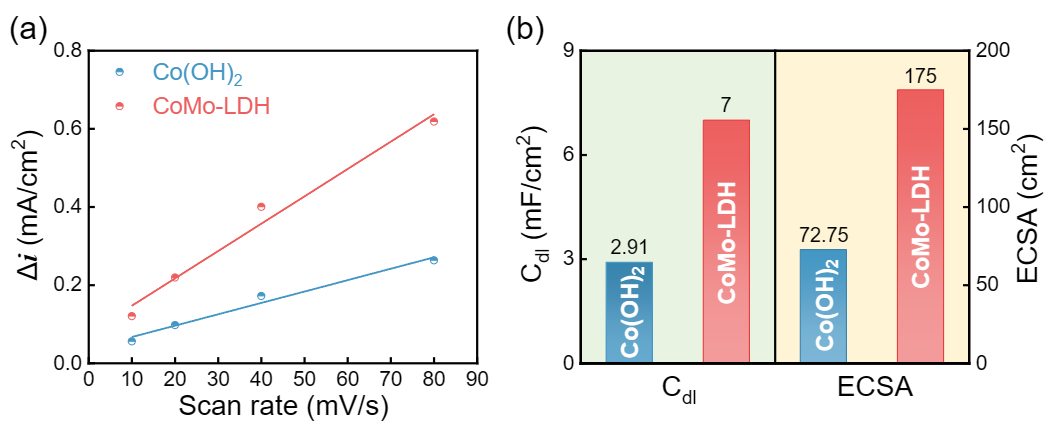


**Figure S13.** Double layer capacitance (a) C_dl_ and (b) electrochemical surface area (ECSA) of Co(OH)_2_ and CoMo-LDH.


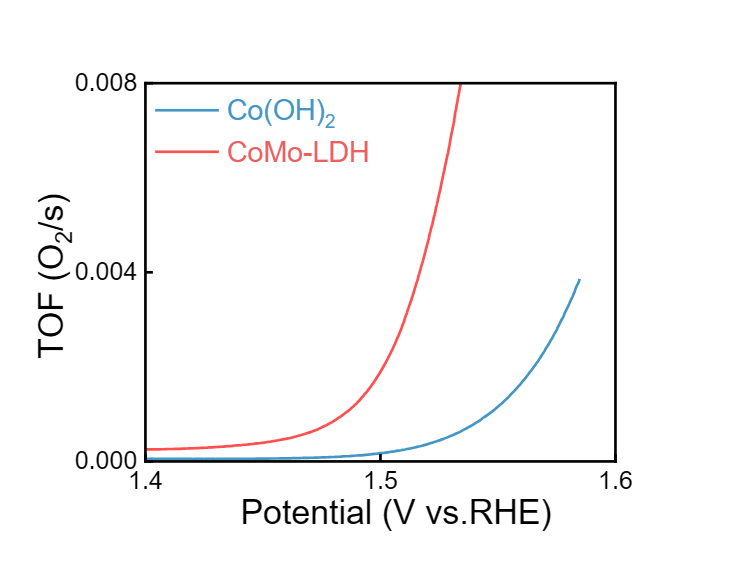


**Figure S14.** Turnover frequency (TOF) of Co(OH)_2_ and CoMo-LDH.


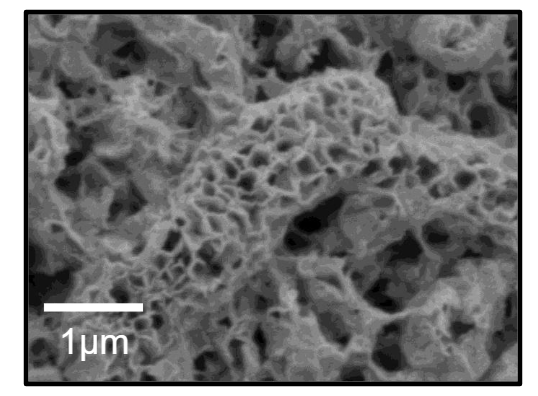


**Figure S15.** SEM image of CoMo-LDH after OER.


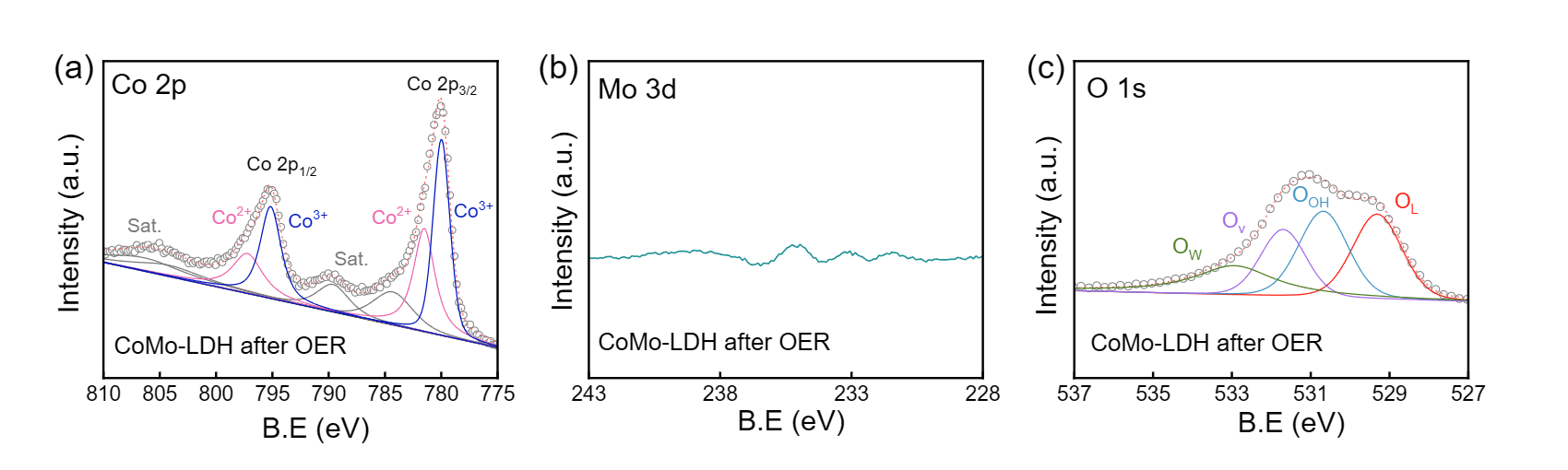


**Figure S16.** High-resolution XPS spectrum of CoMo-LDH after OER: (a) Co 2p, (b) Mo 3d and (c) O 1s.


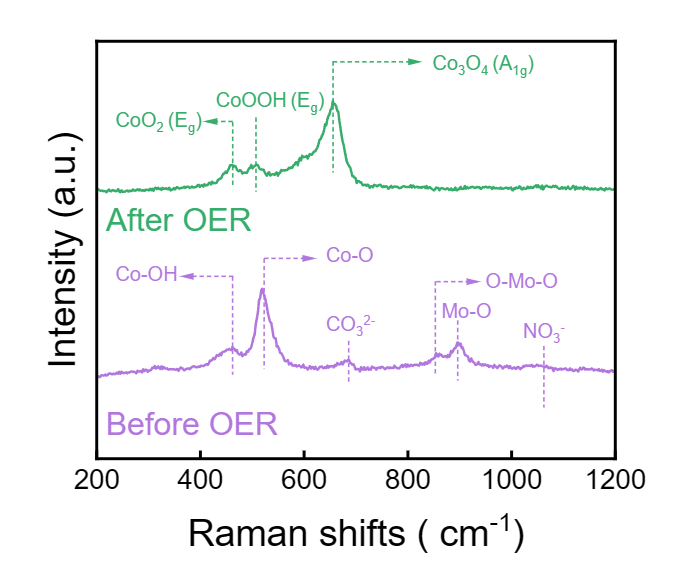


**Figure S17**. Raman spectrum of CoMo-LDH and CoMo-LDH after OER.


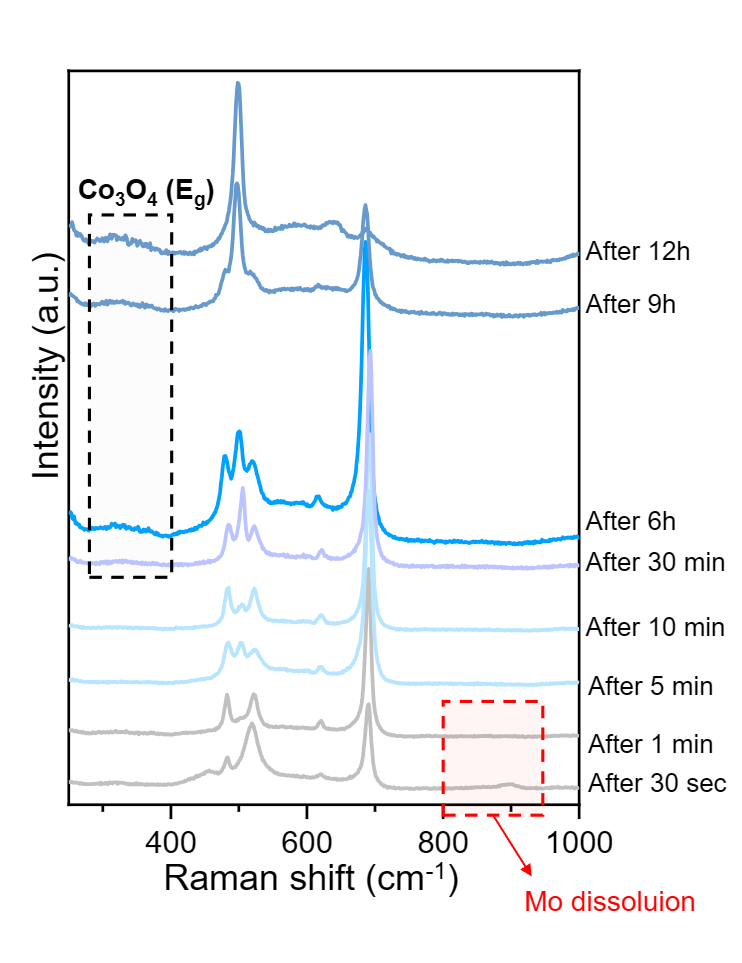


**Figure S18.** Time-resolved in-situ Raman spectra of CoMo-LDH obtained at selected intervals: after 30 sec, 1min, 5min, 30 min, 6h, 9h and up to 12 h, respectively.


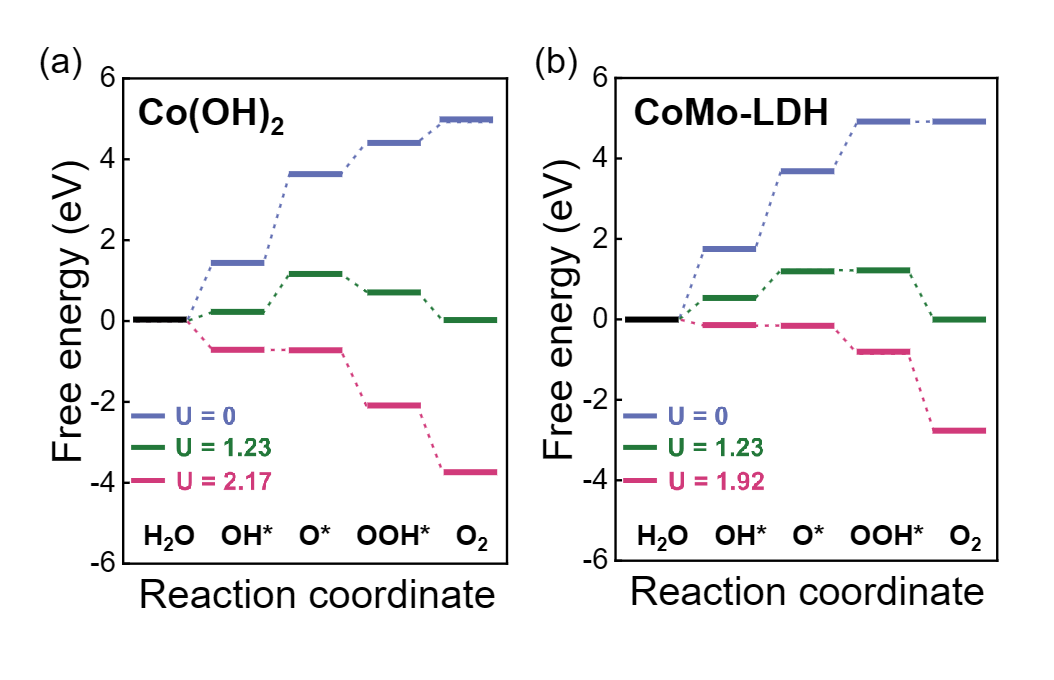


**Figure S19.** The free energy diagram of the (a) Co(OH)_2_ and (b) CoMo LDH at U = 0 V, 1.23 V and their respective limiting potentials.


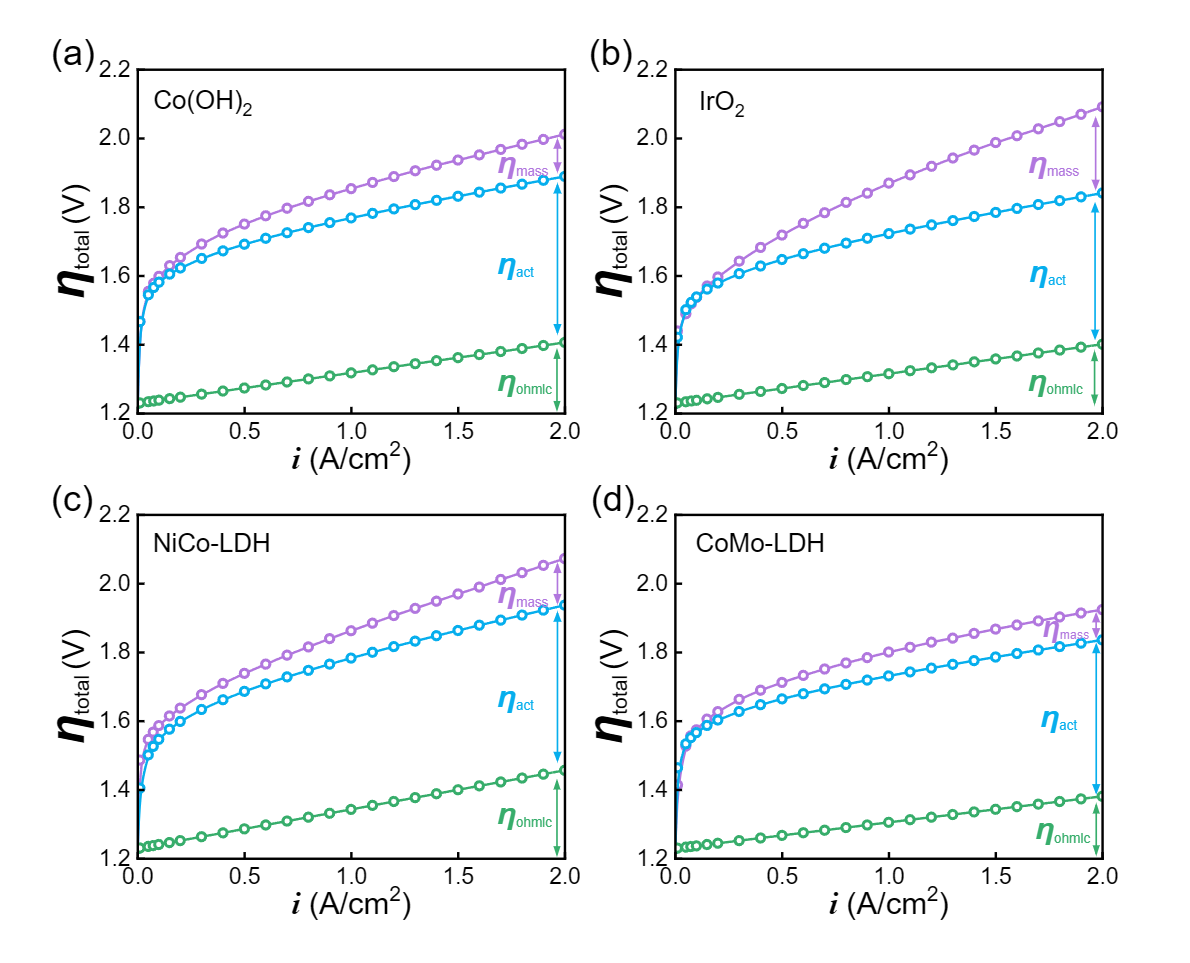


**Figure S20.** Deconvolution of cell voltage. (a) Co(OH)_2_, (b) IrO_2_, (c) NiCo-LDH and (d) CoMo-LDH.


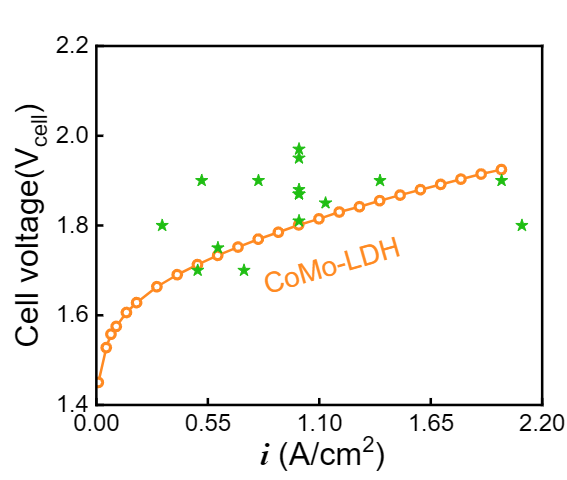


**Figure S21.** Comparison of AEMWE using self-supported based anode in previous researches.


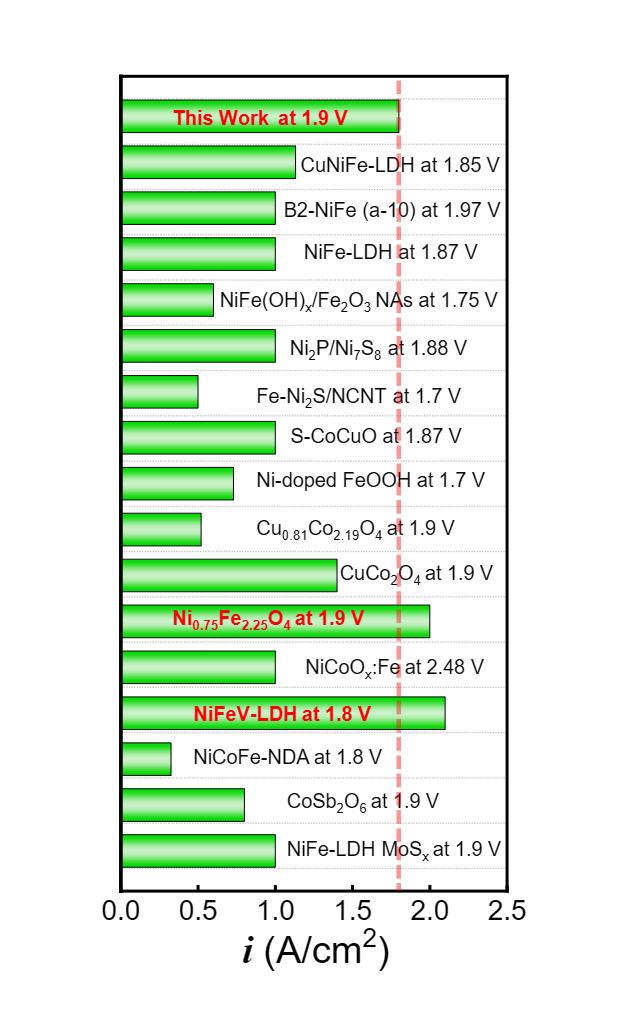


**Figure S22.** Performance comparison of AEMWE using non-PGM based self-supported anodes and Pt/C cathodes.


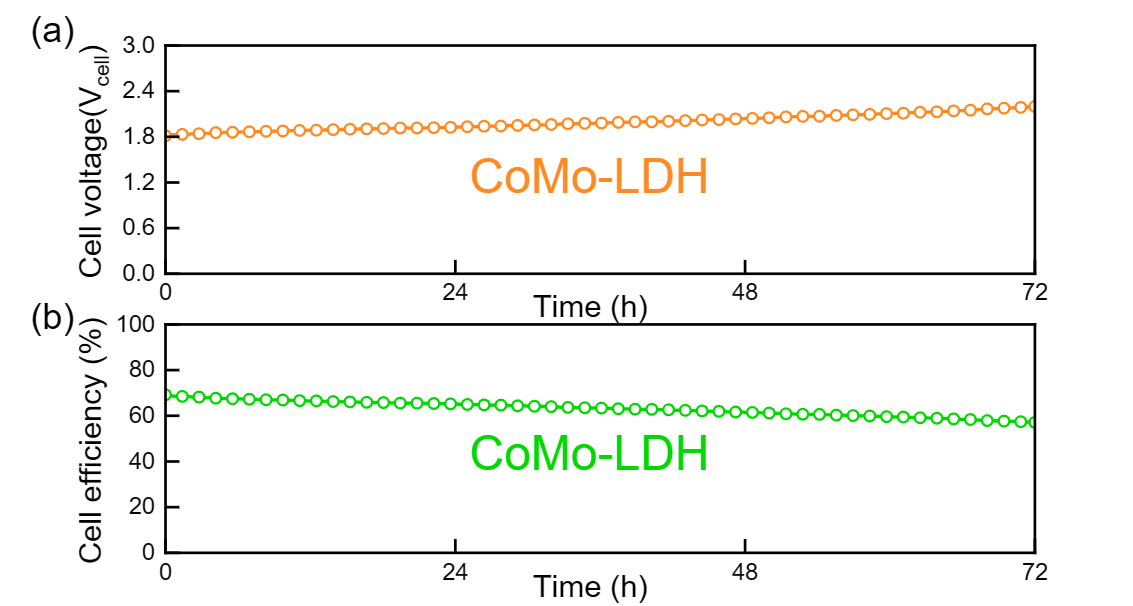


**Figure S23.** (a) Durability test and (b) cell efficiency of AEMWE at 1.0 A/cm^2^ for 72 hours.


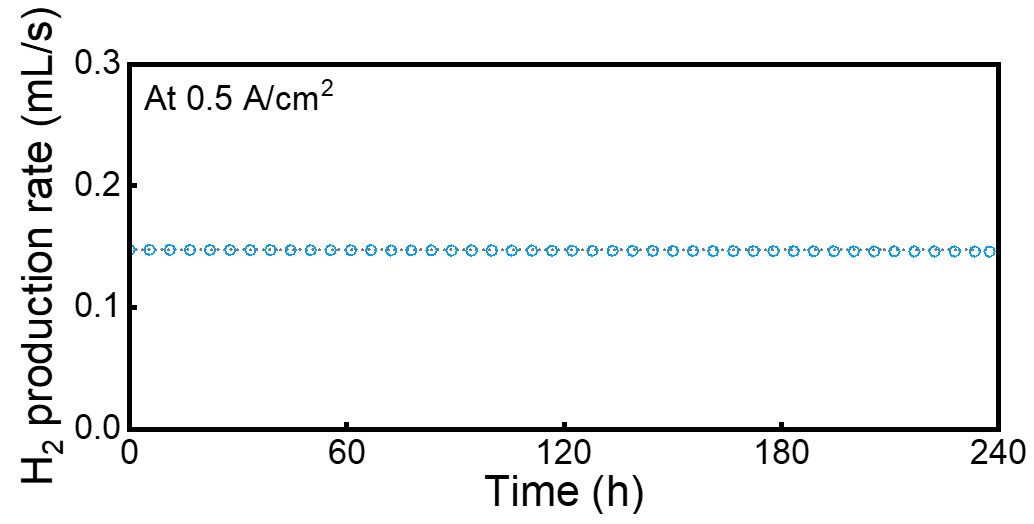


**Figure S24.** H_2_ production rate of AEMWE equipped with CoMo-LDH during durability test at 0.5 A/cm^2^.


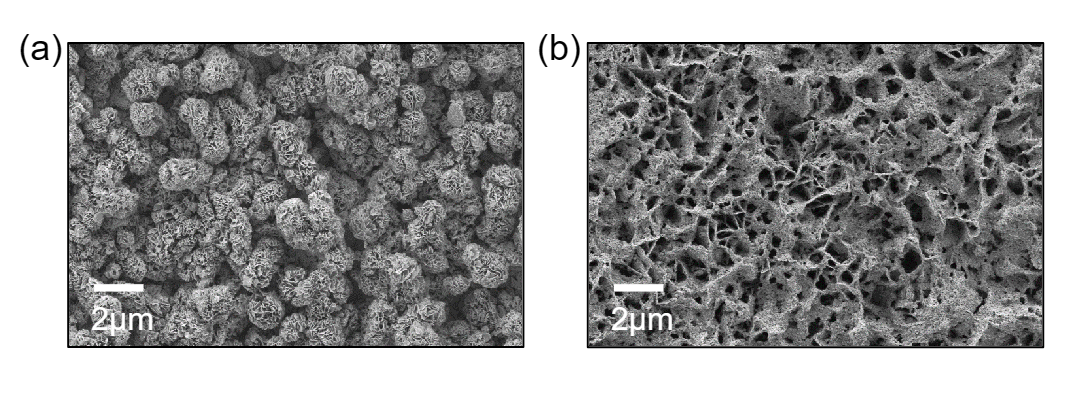


**Figure S25.** SEM images of the CoMo-LDH anode (a) before and (b) after AEMWE durability operation.


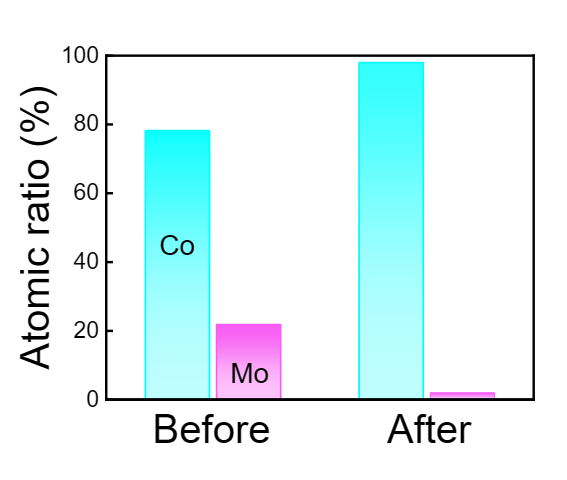


**Figure S26.** Atomic ratios before and after durability test obtained from SEM-EDS analysis.


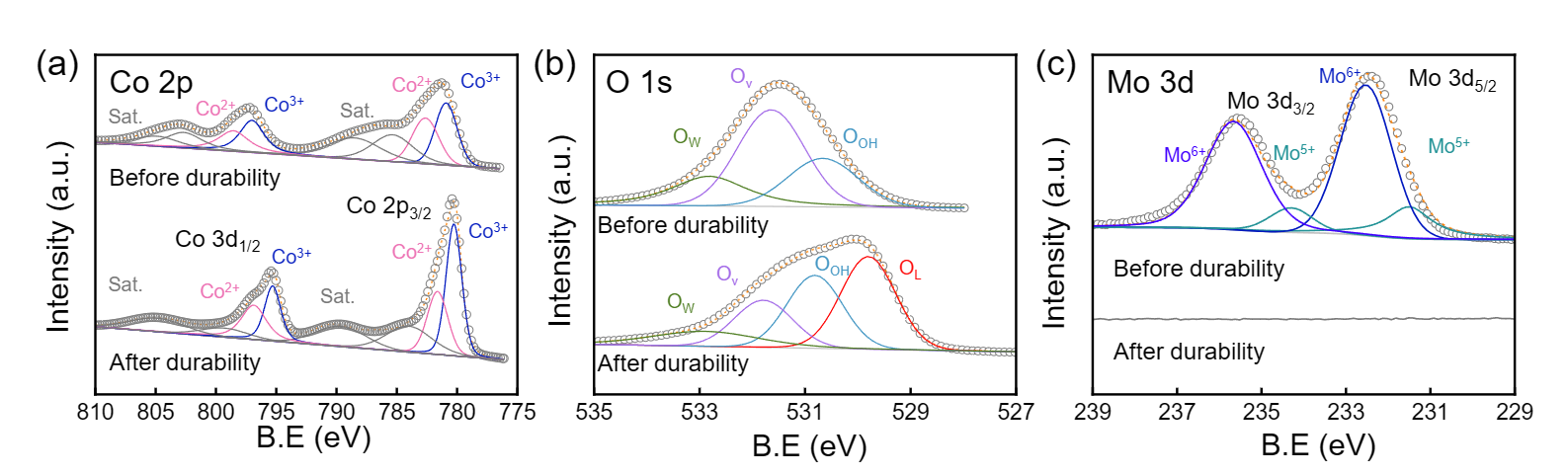


**Figure S27.** High-resolution XPS spectra of CoMo-LDH before and after durability: (a) Co 2p, (b) O 1s (c) Mo 3d.


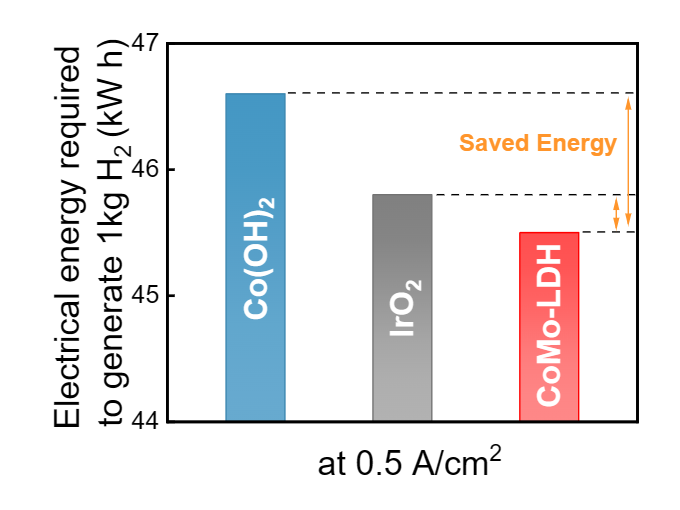


**Figure S28.** Calculation of electricity energy required to generate 1 kg of H_2_ at 0.5 A/cm^2^.

| **Anode** | **Cathode** | **Cell Voltage (V)** | **Current density (A/cm^2^)** | **Electrolyte** | **Temperature** | **Ref.** |
| --- | --- | --- | --- | --- | --- | --- |
| CoMo-LDH | Pt/C | 1.9 | 1.8 | 1 M KOH | 55 | This work |
| CoSb_2_O_6_ | Pt/C | 1.9 | 0.8 | 1 M KOH | 60 | [1] |
| NiCoFe-NDA | Pt/C | 1.8 | 0.325 | 0.1 M KOH | 50 | [2] |
| NiFe-LDH-MoS_x_ | Pt/C | 1.95 | 1 | 1 M KOH | 60 | [3] |
| NiFeV-LDH | Pt/C | 1.8 | 2.1 | 1 M KOH | 50 | [4] |
| Ni_0.75_Fe_2.25_O_4_ | Pt/C | 1.9 | 2 | 1 M KOH | 45 | [5] |
| CuCo_2_O_4_ | Pt/C | 1.9 | 1.4 | 1 M KOH | 45 | [6] |
| Cu_0.81_Co_2.19_O_4_ | Pt/C | 1.9 | 0.52 | 0.1 M KOH | 30 | [7] |
| NiCoO_x_Fe | Pt | 2.48 | 1 | 1 M KOH | 50 | [8] |
| Ni-FeOOH | Pt/C | 1.7 | 0.729 | 1 M KOH+ seawater | 50 | [9] |
| S-CoCuO | Pt/C | 1.87 | 1 | 1 M KOH | 60 | [10] |
| Ni_2_P/Ni_7_S_8_ | Pt/C | 1.88 | 1 | 1 M KOH | 75 | [11] |
| NiFe(OH)_x_/Fe_2_O_3_ NAs | Pt/C | 1.75. | 0.6 | 1 M KOH | 50 | [12] |
| NiFe-LDH | Pt/C | 1.87 | 1 | 1 M KOH | 70 | [13] |
| B2-NiFe-(a-10) | Pt/C | 1.97 | 1 | 1 M KOH | 25 | [14] |
| CuNiFe-LDH | Pt/C | 1.85 | 1.31 | 1 M KOH | 50 | [15] |
| CoCu(OH)_2_ | Pt/C | 1.81 | 1 | 1 M KOH | 55 | [16] |

**Table S1**. Performance of AEMWEs equipped with self-supported based anode.

[1] K. Ham, S. Hong, S. Kang, K. Cho, J. Lee, Extensive Active-Site Formation in Trirutile CoSb2O6 by Oxygen Vacancy for Oxygen Evolution Reaction in Anion Exchange Membrane Water Splitting, ACS Energy Letters, 6 (2021) 364-370.

[2] K. Yue, J. Liu, Y. Zhu, C. Xia, P. Wang, J. Zhang, Y. Kong, X. Wang, Y. Yan, B.Y. Xia, In situ ion-exchange preparation and topological transformation of trimetal–organic frameworks for efficient electrocatalytic water oxidation, Energy & Environmental Science, 14 (2021) 6546-6553.

[3] H. Zhang, G. Shen, X. Liu, B. Ning, C. Shi, L. Pan, X. Zhang, Z.-F. Huang, J.-J. Zou, Self-supporting NiFe LDH-MoSx integrated electrode for highly efficient water splitting at the industrial electrolysis conditions, Chinese Journal of Catalysis, 42 (2021) 1732-1741.

[4] J. Lee, H. Jung, Y.S. Park, S. Woo, J. Yang, M.J. Jang, J. Jeong, N. Kwon, B. Lim, J.W. Han, S.M. Choi, High-Efficiency Anion-Exchange Membrane Water Electrolyzer Enabled by Ternary Layered Double Hydroxide Anode, Small, 17 (2021) 2100639.

[5] J. Lee, H. Jung, Y.S. Park, S. Woo, N. Kwon, Y. Xing, S.H. Oh, S.M. Choi, J.W. Han, B. Lim, Corrosion-engineered bimetallic oxide electrode as anode for high-efficiency anion exchange membrane water electrolyzer, Chemical Engineering Journal, 420 (2021) 127670.

[6] Y.S. Park, M.J. Jang, J. Jeong, S.M. Park, X. Wang, M.H. Seo, S.M. Choi, J. Yang, Hierarchical Chestnut-Burr Like Structure of Copper Cobalt Oxide Electrocatalyst Directly Grown on Ni Foam for Anion Exchange Membrane Water Electrolysis, ACS Sustainable Chemistry & Engineering, 8 (2020) 2344-2349.

[7] W.-S. Choi, M.J. Jang, Y.S. Park, K.H. Lee, J.Y. Lee, M.-H. Seo, S.M. Choi, Three-Dimensional Honeycomb-Like Cu0.81Co2.19O4 Nanosheet Arrays Supported by Ni Foam and Their High Efficiency as Oxygen Evolution Electrodes, ACS Applied Materials & Interfaces, 10 (2018) 38663-38668.

[8] D. Xu, M.B. Stevens, M.R. Cosby, S.Z. Oener, A.M. Smith, L.J. Enman, K.E. Ayers, C.B. Capuano, J.N. Renner, N. Danilovic, Y. Li, H. Wang, Q. Zhang, S.W. Boettcher, Earth-Abundant Oxygen Electrocatalysts for Alkaline Anion-Exchange-Membrane Water Electrolysis: Effects of Catalyst Conductivity and Comparison with Performance in Three-Electrode Cells, ACS Catalysis, 9 (2019) 7-15.

[9] Y.S. Park, J. Lee, M.J. Jang, J. Yang, J. Jeong, J. Park, Y. Kim, M.H. Seo, Z. Chen, S.M. Choi, High-performance anion exchange membrane alkaline seawater electrolysis, Journal of Materials Chemistry A, 9 (2021) 9586-9592.

[10] J. Zhang, S. Zhao, B. Chen, S. Yin, Y. Feng, Y. Yin, Sulfidation of CoCuOx Supported on Nickel Foam to Form a Heterostructure and Oxygen Vacancies for a High-Performance Anion-Exchange Membrane Water Electrolyzer, ACS Applied Materials & Interfaces, 15 (2023) 45756-45763.

[11] F.-L. Wang, N. Xu, C.-J. Yu, J.-Y. Xie, B. Dong, X.-Y. Zhang, Y.-W. Dong, Y.-L. Zhou, Y.-M. Chai, Porous heterojunction of Ni2P/Ni7S6 with high crystalline phase and superior conductivity for industrial anion exchange membrane water electrolysis, Applied Catalysis B: Environmental, 330 (2023) 122633.

[12] H. Wang, H. Sun, S. Cao, Y. Wang, X. Du, J. Li, Amorphous-crystalline interface coupling induced highly active ultrathin NiFe oxy-hydroxide design towards accelerated alkaline oxygen evolution, Journal of Catalysis, 430 (2024) 115354.

[13] D. Guo, J. Chi, H. Yu, G. Jiang, Z. Shao, Self-Supporting NiFe Layered Double Hydroxide “Nanoflower” Cluster Anode Electrode for an Efficient Alkaline Anion Exchange Membrane Water Electrolyzer, Energies, 2022.

[14] Y. Ma, J.-J. Wang, X.-H. Liu, N. Xu, X. Li, Y.-H. Wang, L.-M. Zhao, Y.-M. Chai, B. Dong, Short-time potentiostatic assisted borate to induce the generation of ultrathin NiFe LDH active phase for industrial-level water oxidation, Chemical Engineering Journal, 490 (2024) 151490.

[15] D. Chanda, H. Kwon, M.M. Meshesha, J.S. Gwon, M. Ju, K. Kim, B.L. Yang, Modulating interfacial electronic coupling of copper-mediated NiFe layered double hydroxide nanoprisms via structural engineering for efficient OER in wireless photovoltaic-coupled and anion exchange membrane water electrolysis, Applied Catalysis B: Environmental, 340 (2024) 123187.

[16] S.H. Park, S.H. Lee, J.-Y. Jeong, H. Jin, J.S. Ha, S.J. Lee, I.T. Kim, C. Kim, S. Kim, M. Bae, H. Lee, S.M. Choi, k. Yangdo, Y.S. Park, Three-dimensional copper cobalt hydroxide electrode for anion exchange membrane water electrolyzer, International Journal of Hydrogen Energy, 48 (2023) 29877-29886.
